# Cavitation-regime-controlled synergy in high-frequency sonophotocatalysis

**DOI:** 10.1016/j.ultsonch.2026.107996

**Published:** 2026-08-05

**Authors:** Vincenzo Fabbrizio, Melissa G. Galloni, Ermelinda Falletta, Enrique Rodriguez Castellon, Giuseppina Cerrato, Fabio Gosetti, Claudia L. Bianchi

**Affiliations:** a Dipartimento di Chimica, Università degli Studi di Milano, via Golgi 19, 20133 Milano, Italy; b Consorzio Interuniversitario Nazionale per la Scienza e Tecnologia dei Materiali (INSTM), Via Giusti 9, 50121 Firenze, Italy; c Department of Inorganic Chemistry, Crystallography, and Mineralogy, Faculty of Sciences, Instituto Interuniversitario de Investigación en Biorrefinerías I3B, University of Malaga, 29071 Málaga, Spain; d Dipartimento di Chimica, Università degli Studi di Torino, Via Giuria 7, 10125 Torino, Italy; e Department of Earth and Environmental Sciences, Università degli Studi Milano-Bicocca, Milano, Italy

**Keywords:** Process intensification, Plate-type ultrasonic reactor, Synergy, Bismuth oxyhalides, Titanium dioxide, Ibuprofen, Ultrasound

## Abstract

High-frequency ultrasound is proposed as an intensification tool for advanced oxidation processes, yet its role is often treated as a simple energy input and not as a regime-dependent process variable. This work investigated reactor-driven synergy in sonophotocatalysis using a plate-type ultrasonic system operating at 584 kHz for ibuprofen (IBU) degradation.

Cavitation efficiency was quantified by potassium iodide dosimetry, identifying 584 kHz in continuous mode as the most effective and stable radical-generating condition. The individual and combined effects of ultrasound and simulated solar irradiation were evaluated using BiOCl, BiOBr, and TiO_2_ P25.

True synergy emerged only for BiOBr. The kinetic constant of the coupled process (4.55·10^-2^ min^−1^) exceeded the sum of the individual photocatalytic and sonocatalytic contributions by a factor of 2.23, corresponding to *ca.* 55% synergy. Reactive-species trapping indicated a cooperative mechanism involving photogenerated holes together with cavitation-derived hydroxyl and superoxide radicals. The synergistic contribution was retained in simulated drinking water and at reduced catalyst loading.

Sonophotocatalysis also enhanced TOC removal relative to the individual processes, although mineralization remained partial, indicating sequential oxidation under the investigated conditions. Transformation products were identified by UHPLC–MS/MS and their potential ecotoxicological relevance was assessed through ECOSAR modelling.

A preliminary process-oriented assessment yielded a cost of ownership of *ca.* 2.20 €·m^−3^ and an electrical energy per order of 0.49 kWh·m^−3^·order^-1^ for 90% IBU abatement.

These findings demonstrate that synergy is not intrinsic to process coupling, but depends on the cavitation regime and photocatalyst properties, supporting reactor-oriented development of high-frequency sonophotocatalytic systems.

## Graphical abstract

**Figure:**
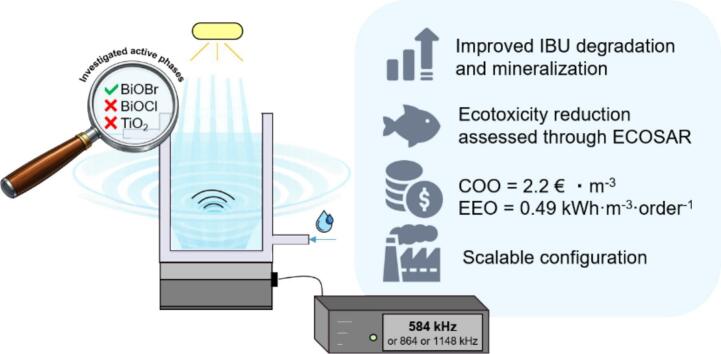

## 1 Introduction

Over the past decades, ultrasound-assisted processes have gained increasing attention in environmental remediation, mainly due to the unique physicochemical effects generated by acoustic cavitation [1], [2], [3], [4]. The collapse of cavitation bubbles creates localized high temperatures and pressures, intense shear forces, and short-lived radical species, giving rise to highly reactive microenvironments that cannot be reproduced under conventional hydrodynamic conditions [5], [6], [7]. Since the pioneering work in sonochemistry [8], [9], it has become clear that cavitation cannot be considered as a single, uniform phenomenon, but as a regime-dependent process strongly influenced by acoustic frequency, power density, reactor geometry and liquid properties [7], [10]. This process is associated with violent and transient cavitation events, often dominated by mechanical effects, such as microjet formation and erosion [11]. Conversely, high-frequency configurations tend to produce more spatially homogeneous cavitation fields, where chemical effects and radical generation play a more prominent role [12], [13], [14], [15], [16]. Despite this well-recognized regime dependence, ultrasound is still frequently treated as a fixed energy input, rather than as a tuneable process variable. As a consequence, the influence of reactor design and operating frequency on reaction pathways and catalytic performance is often not systematically addressed, especially in view of process reproducibility and potential scale-up [7], [10], [17].

These aspects become even more critical when ultrasound is coupled with photocatalysis. Sonophotocatalysis combines cavitation-induced radical formation with photoactivated surface reactions and is generally proposed as an intensification strategy capable of improving mass transfer, mitigating charge recombination, and enhancing overall oxidation efficiency [18], [19], [20]. Although many studies report higher degradation rates under simultaneous ultrasonic and light irradiation compared to the individual processes, the existence of true synergy is not always rigorously demonstrated [21], [22], [23]. In several cases, the observed improvement can be rationalized as the simple sum of the independent contributions of sonolysis and photocatalysis, while quantitative approaches able to discriminate additive from cooperative effects remain relatively scarce.

At the same time, research in sonophotocatalysis has largely focused on catalyst design, including doping strategies, heterojunction construction, and surface modification [24]. While material engineering plays a central role, less attention has been devoted to how the imposed cavitation regime itself influences the behaviour of the combined system [25]. Considering that cavitation dynamics directly govern radical formation, bubble collapse characteristics, and transport phenomena at the solid–liquid interface, the way in which ultrasound is delivered into the reactor is expected to critically modulate the interaction between ultrasonic and photocatalytic pathways [26].

High-frequency plate-type ultrasonic systems represent an interesting configuration in this respect. Compared to conventional horn reactors, they generate distinct cavitation conditions, typically characterized by more uniform acoustic fields and predominantly chemical effects [27]. However, systematic studies that directly link cavitation characterization, quantitative kinetic analysis, and catalyst-dependent response under high-frequency operation are still limited.

From the materials perspective, semiconductor photocatalysts with different electronic structures provide a useful framework to separate intrinsic catalytic properties from reactor-driven effects. Titanium dioxide (TiO_2_ P25) is the benchmark material in environmental photocatalysis, although its wide band gap limits activation mainly to the UV region [28], [29], [30], [31]. In contrast, bismuth oxyhalides (BiOX, where X = Cl, Br) are layered semiconductors characterized by an internal electric field and tuneable band gaps, enabling progressively enhanced visible-light absorption from BiOCl to BiOBr [32], [33], [34], [35]. These differences make BiOCl and BiOBr suitable candidates to investigate how high-frequency cavitation-assisted radical generation interacts with photocatalytic charge carriers under simulated solar irradiation.

In this work, high-frequency sonophotocatalysis at 584 kHz was investigated using a plate-type ultrasonic reactor for the degradation of ibuprofen (IBU) as a model emerging contaminant. The cavitation regime imposed by the reactor configuration was first characterized by potassium iodide (KI) dosimetry to identify the most effective radical-generating conditions. The individual photo- and sonochemical processes were then evaluated as reference systems, before assessing the combined sonophotocatalytic performance of BiOCl, BiOBr and TiO_2_ P25. Synergy was quantitatively determined through kinetic analysis by comparing the rate constant of the coupled process with the sum of the individual contributions.

Process robustness was further examined by varying catalyst loading and water matrix composition, while mineralization was assessed through total organic carbon (TOC) measurements. Transformation products formed during degradation were subsequently identified by UHPLC–MS/MS analysis, and their potential ecotoxicological relevance was assessed through ECOSAR modelling combined with principal component analysis.

Finally, a preliminary process-oriented evaluation was carried out to contextualize the experimental findings within a broader technological framework.

Through this reactor-centered approach, the present study aims to clarify how cavitation regimes imposed by high-frequency plate-type ultrasonic reactors govern the emergence, magnitude, and reproducibility of synergistic effects in sonophotocatalytic systems.

## 2 Materials and methods

### 2.1 Materials and characterisation

Details on materials used and characterisation techniques are reported in Supplementary Information (SI).

### 2.2 Materials preparation

Commercial titanium dioxide P25 (TiO_2_ P25) was used as received, whereas bismuth oxyhalides (BiOX, X = Cl or Br) were synthesized *via* a conventional co-precipitation. Following the procedure recently reported [34], [35], [36], *ca.* 1 g each BiOX was obtained by dropwise addition of 33 mL of a 0.1 M KX (X = Cl, Br) aqueous solution to 55 mL of a 0.1 M Bi(NO_3_)_3_·5H_2_O solution prepared in 9% v/v aqueous acetic acid. The addition was carried out under continuous magnetic stirring (250 rpm). During the process, a white precipitate formed for BiOCl, while a pale-yellow precipitate was observed for BiOBr. The resulting suspensions were stirred at room temperature for 24 h. Upon completion of the reaction, the solid products were recovered by vacuum filtration using a Büchner funnel, washed with deionized water until neutral pH was reached, and finally dried at 120 °C overnight.

### 2.3 High-frequency sonophotocatalytic tests for ibuprofen abatement

#### 2.3.1 Reactor configuration and operating conditions

**Fig. 1:**
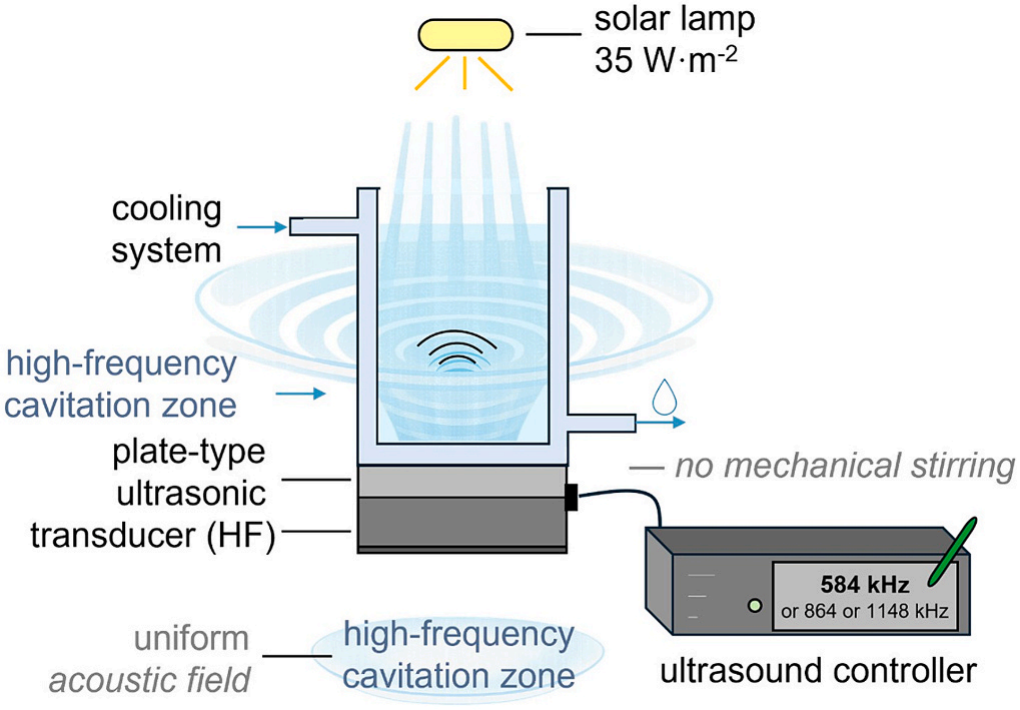
Schematic representation of the high-frequency plate-type ultrasonic reactor used for sonophotocatalytic experiments.

High-frequency sonophotocatalytic experiments were carried out using a plate-type ultrasonic reactor (Meinhardt Ultrasonics) equipped with a multifrequency generator operating at 584, 864, and 1148 kHz. Ultrasound was delivered from the bottom through a vibrating plate transducer, generating a spatially distributed acoustic field across the reactor volume. A schematic representation of the reactor configuration employed in this study is shown in Fig. 1.

The reactor consisted of a bottomless jacketed glass vessel with a working volume of 300 mL, mounted directly on the ultrasonic transducer connected to an ultrasound multifrequency generator (Meinhardt Ultrasonics), producing different ultrasound frequencies (584, 864, 1148 kHz) at constant amplitude (50%) and in pulse/continuous mode. Irradiation was provided from the top by a simulated solar lamp (300 W Ultra-Vitalux OSRAM), delivering an irradiance of 35 W·m^−2^ at the reactor surface. All experiments were conducted inside a custom-made dark box to eliminate interference from ambient light. The reaction temperature was maintained at 21.0 ± 0.1 °C throughout all tests.

#### 2.3.2 Potassium iodide (KI) dosimetry

KI dosimetry was used to evaluate the cavitation efficiency of the high-frequency ultrasonic reactor at different operating frequencies (584, 864, 1148 kHz). This method allows indirect quantification of hydroxyl radicals (HO·) generated during sonolysis through the oxidation of iodide ions (I^-^) to triiodide ions (I_3_^-^) [11], [16], [37].

Dosimetry experiments were carried out using 300 mL of a 0.1 M KI solution in ultrapure or simulated drinking water (prepared according to Table S.1 [38]) at spontaneous pH, under atmospheric conditions and in the absence of light. Ultrasound was applied in pulse (3 s on/ 2 s off) or continuous mode at selected frequencies (584, 864, and 1148 kHz), while the amplitude was kept constant at 50%. Experiments were conducted for 30 min, and aliquots were withdrawn every 5 min, filtered with 0.22 μm nylon filters, and analysed by UV–vis spectroscopy (Shimadzu UV-2600i UV–vis spectrophotometer) by monitoring the absorbance of I_3_^-^ at 355 nm (ε = 26 303 L·mol^−1^·cm^−1^). The evolution of I_3_^-^ concentration along with time was used as comparative indicator of radical generation efficiency under different acoustic conditions [39].

#### 2.3.3 Ibuprofen abatement tests

Details are reported in SI.

#### 2.3.4 Transformation products (TPs) identification

When BiOBr was used as catalyst, aliquots of degraded IBU solutions were subjected to ultra-high performance liquid chromatography coupled to tandem mass spectrometry analysis (UHPLC-MS/MS) after proper dilution. The UHPLC analyses were carried out by Nexera 40 (Shimadzu, Kyoto, Japan) coupled with the QTRAP 6500 + mass spectrometer (Sciex, Framingham, USA) through an electrospray ionization (ESI) source. Instrument control and data acquisition were performed by Analyst 1.7.3 software (Sciex, Framingham, USA), whereas data reprocessing was carried out by Sciex OS 3.3.1 (Sciex, Framingham, USA).

The stationary phase used was a Kinetex XB-C18 column (100 x 3 mm, 1.7 µm; Phenomenex, Torrence, CA, USA). The mobile phase was a mixture of water (A) and acetonitrile (B), both added with formic acid 0.1% (v/v). The mobile phase flow rate was 400 μL·min^−1^ eluting in gradient mode as follows: 0.0–1.0 min, 5% solvent B; 10.0 min, 100% solvent B; 10.0–12.0 min, 100% solvent B; 12.1 min, 5% solvent B; 12.1––15.0 min, 5% solvent B. The injection volume was 10 μL, and the column oven temperature was set at 35 °C.

The ESI source worked in negative ion mode. The instrumental parameters were set as follows: curtain gas (N_2_) at 40 psig, both ion source gases GS1 (air) and GS2 (air) at 60 psig, desolvation temperature (TEM) at 450 °C, collision activated dissociation gas (CAD) at 12 units of the arbitrary scale of the instrument, ion spray voltage (IS) at −4500 V, declustering potential (DP) at −16 V, entrance potential (EP) at −10 V, collision energy (CE) and collision energy spread in Enhanced Product Ion mode at −45 V and ± 15 V, respectively. The mass spectrometer was used in data-dependent acquisition mode, using the third quadrupole as an ion trap. The first experiment consisted in a non-target screening using Enhanced MS (EMS, full scan mode) as a survey scan over the mass range 100–450 u. When the signal of a detected compound exceeds a defined threshold, this survey scan automatically triggers the acquisition of both Enhanced Resolution (ER) to confirm the isotope ratio, and Enhanced Product Ion spectrum (EPI) to obtain the MS/MS fingerprint. In this way, for each detected compound, it is possible to perform a second experiment after the selection of a precursor ion and at least two product ions to build a Multiple Reaction Monitoring (MRM) method used as a survey scan and followed by an EPI spectrum to confirm the species of interest.

Unit mass resolution was established and maintained in each mass-resolving quadrupole by keeping a full width at half maximum (FWHM) of about 0.7 u.

#### 2.3.5 In silico assessment of transformation product toxicity

The estimation of aquatic toxicity of transformation products (TPs) was performed *in silico* using ECOSAR software (US EPA) [40]. The program estimates both acute and chronic toxicity to aquatic organisms, such as fish, invertebrates, and plants, by using computerized Structure Activity Relationships (SARs).

#### 2.3.6 Economic assessment

A preliminary economic assessment was carried out to evaluate the high-frequency plate-type ultrasonic reactor (Meinhardt Ultrasonics) from an operational and energy-efficiency perspective under optimized experimental conditions identified in this study (sonophotocatalytic process using BiOBr at 0.5 g·L^−1^ for the abatement of IBU at 50 mg·L^−1^).

The evaluation was designed as a preliminary analysis aimed at framing the experimental findings.

The economic evaluation focused on the Meinhardt configuration operating under the optimized conditions reported in the experimental section. A reference treatment capacity of 1 m^3^·h^−1^ and an annual operating time of 6000 h were assumed. Annual costs were divided into capital expenditure (CAPEX) and operating expenditure (OPEX). CAPEX was estimated considering the main system components, including ultrasonic reactor, generator, light source, cooling system, and auxiliary equipment and was annualized using a capital recovery factor, assuming a plant lifetime of 5 years and constant operating conditions. OPEX included electricity consumption, routine maintenance, and catalyst (BiOBr) replacement. Electrical energy demand was calculated based on the total electrical power demand of the ultrasonic reactor, irradiation source, and cooling system under the selected operating conditions. Catalyst consumption was estimated assuming a dosage of 0.5 kg·m^−3^ and a conservative annual loss of 5%.

To relate economic performance to process efficiency, the analysis was complemented by the calculation of the cost of ownership (COO, eq. (1), defined as the annualized cost of the treatment system [10]. This figure of merit [41] is commonly used for the comparative evaluation of advanced oxidation processes [42], [43], [44], [45].

(1) $$COO\;\left(€\cdot m^{-3}\right)=\;\frac{total\;annual\;cost\;}{production\;unit\;\cdot \;annual\;work\;time}$$

The estimated COO was contextualized against representative cavitation-based treatment technologies reported in the literature [10], while acknowledging differences in reactor scale, operating conditions, treatment objectives, and cost boundaries.

In addition to the cost-of-ownership calculation, the electrical energy per order (EEO) was calculated to relate the electrical energy demand to IBU abatement performance. EEO was calculated according to:

(2) $$EEO\left(kWh\cdot m^{-3}\cdot {\mathrm{order}}^{-1}\right)=\frac{P_{\mathrm{tot}}\cdot t}{V\cdot \log(\frac{C_{0}}{C_{t}})}$$

where *P_tot_* is the total electrical power demand of the ultrasonic reactor, irradiation source, and cooling system (kW), *t* is the treatment time (h), *V* is the treated water volume (m^3^), *C_0_* and *C_t_* are the initial and final IBU concentrations, respectively. For comparison purposes, the EEO value was calculated at 90% IBU abatement, corresponding to one order of magnitude reduction. Since mineralization was only partial under the investigated conditions, EEO was not extrapolated to complete TOC removal. For the optimized BiOBr-assisted sonophotocatalytic process, t_90_ was calculated from the pseudo-first-order kinetic constant (k = 4.55 ·10^-2^ min^−1^), giving t_90_ = 50.6 min. Considering the total electrical power demand of 0.58 kW, this resulted in an EEO of 0.49 kWh·m^−3^·order^-1^ for 90% IBU abatement. Therefore, the reported EEO should be considered as an energy-performance metric for parent-compound abatement and not as the energy requirement for complete mineralization.

## 3 Results and discussion

High-frequency cavitation is characterized by the formation of a spatially distributed population of transient bubbles, typically associated with enhanced radical generation and limited mechanical erosion [46]. Under these conditions, oxidation processes are expected to be predominantly chemically driven, making high-frequency operation particularly suitable for aqueous pollutant degradation [46].

Here, high-frequency ultrasound (584 kHz) was coupled with photocatalysis for the abatement of ibuprofen (IBU). To distinguish intrinsic catalytic properties from the cavitation effects imposed by the reactor, three semiconductor materials were investigated: two bismuth oxyhalides, in particular BiOCl and BiOBr, selected for their layered structures and visible-light response [47], and TiO_2_ P25 as a benchmark reference [48].

The comparative approach allows decoupling material-dependent behaviour from cavitation-regime effects, providing a framework to evaluate whether synergy arises from catalyst engineering or from the imposed high-frequency reactor configuration.

### 3.1 Physico-chemical properties of materials

The structural and surface properties of the investigated materials were analyzed to establish a defined baseline for interpreting their behavior under high-frequency sonophotocatalytic conditions. The main physico-chemical properties are summarized in Table 1.

**Table 1:** Main properties of BiOX compared to TiO_2_ P25.

Code	surface area^a^ (m^2^·g^−1^)	pH_pzc_^b^	bandgap^c^ (eV)	valence band^d^ (V *vs.* RHE)
TiO_2_ P25	46	6.5^e^	3.19^f^	2.90
BiOCl	13	4.46	3.34	2.31
BiOBr	10	5.02	2.63	2.19

a by BET equation (2)-parameters); ^b^from procedure described in Ref [49]; ^c^by Tauc plot; ^d^from VB to XPS; ^e^from Ref. [50]; ^f^from Ref. [51].

**Fig. 2:**
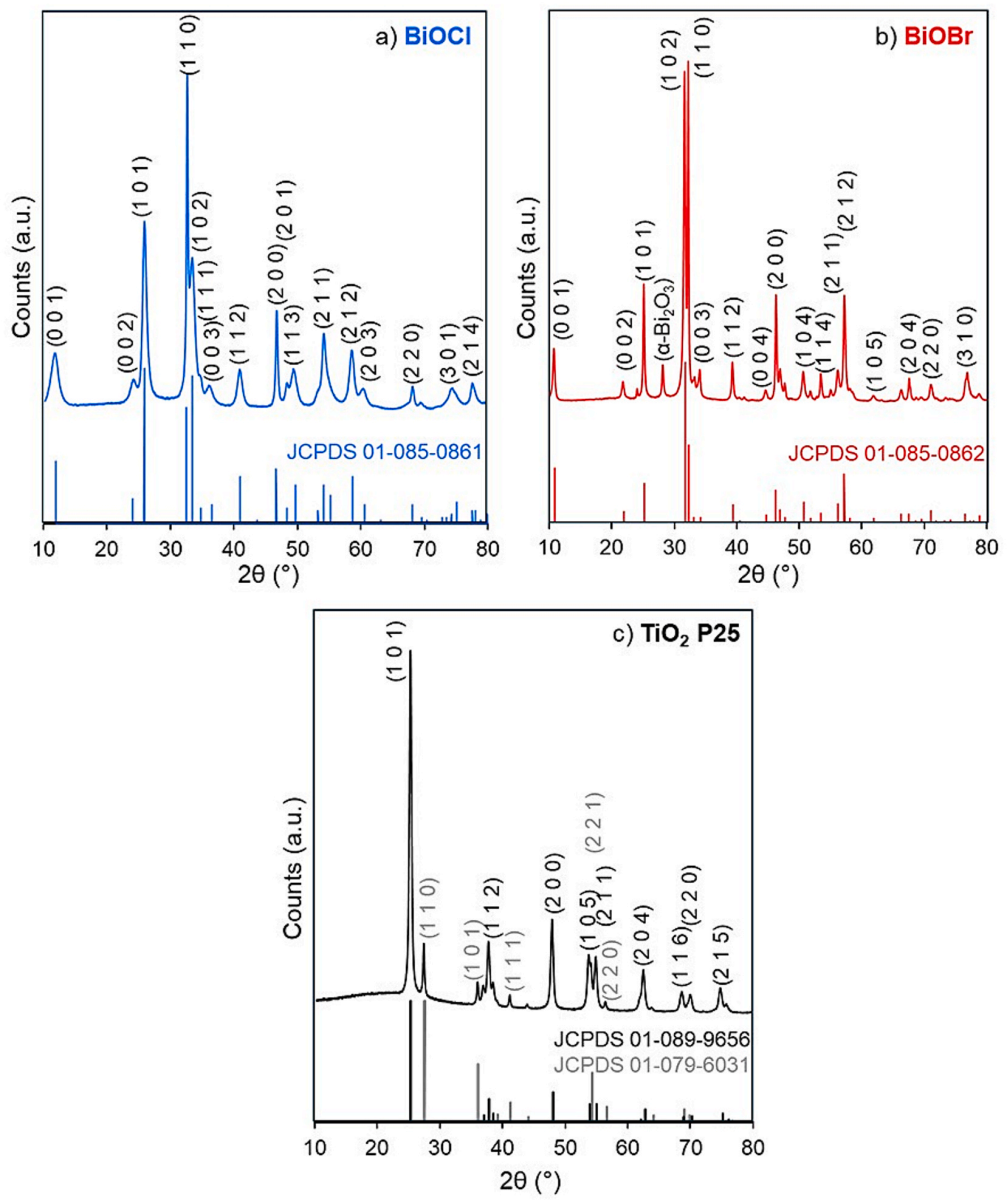
XRD patterns of BiOCl, BiOBr and TiO_2_ P25 compared to the related references.

XRD patterns (Fig. 2) confirm the formation of crystalline BiOCl and BiOBr phases [52], [53], while TiO_2_ P25 exhibits the expected anatase–rutile biphasic composition [54]. In the case of BiOBr, a weak additional reflection attributable to trace α-Bi_2_O_3_ was detected, although its low intensity suggests a negligible impact on catalytic performance [53]. Generally, the diffraction results indicate good crystallinity and phase purity for all investigated materials.

FESEM images (Fig. S.1a,b) reveal the typical layered morphology of bismuth oxyhalides, consisting of lamellar nanosheets assembled into flower-like aggregates [22], [55]. BiOBr displays a comparatively more open and loosely packed structure, whereas BiOCl appears more compact. In both cases nanosheets exhibit an almost comparable thickness in the 20–40 nm with a different external appearance: slightly more ordered terminations of the nanosheets are observed in the case of the BiOCl material (see the inset to Fig. S.1a) with respect of those of the BiOBr system, for which more “damaged” contours are evident (see the inset to Fig. S.1.b).

In contrast, as well-known from the literature [56], [57], TiO_2_ P25 is composed of aggregated quasi-spherical nanoparticles, consistent with its well-known morphology. These morphological differences may influence interfacial accessibility and mass transport under dynamic reaction conditions. Under ultrasonic irradiation, the open flower-like assembly of BiOBr nanosheets may provide a more accessible solid–liquid interface and facilitate liquid renewal within the inter-sheet regions. Conversely, the more compact BiOCl aggregates may provide less accessible inter-sheet pathways for acoustic microstreaming and convective transport. In the present high-frequency reactor, these effects are expected to arise mainly from acoustic streaming and local mixing rather than from pronounced particle erosion or microjet-induced surface damage. Although no direct hydrodynamic measurements were performed, morphology-dependent interfacial transport may therefore contribute, together with the different electronic structures, to the distinct response of the two BiOX materials under dual irradiation.

Textural properties, determined by N_2_ adsorption–desorption isotherms (Fig. S.1c-e), show relatively low surface areas for BiOCl and BiOBr (10–13 m^2^·g^−1^, Table 1), typical of layered materials synthesized *via* precipitation routes [22]. TiO_2_ P25 exhibits a higher surface area (*ca.* 46 m^2^·g^−1^, Table 1), in agreement with its nanosized particle structure [58].

However, as discussed in the following sections, the sonophotocatalytic performances under high-frequency cavitation conditions do not directly correlate with surface area alone, suggesting that electronic structure and reactor-imposed effects play a more relevant role.

Surface composition of BiOCl and BiOBr was investigated by XPS to verify oxidation state and surface stoichiometry. Survey spectra (Figs. S.2 and S.3) confirm the presence of Bi, O and the corresponding halide (Cl or Br), with no detectable impurities.

High-resolution spectra of the Bi *4f*, O *1s*, and Cl *2p*, Br *3d* regions are fully consistent with the expected chemical states of Bi^3+^, lattice oxygen and halide ions incorporated within the BiOX structure [59]. In both materials, the O *1s* region (Figs. S.2c and S.3c) shows minor contributions attributable to surface hydroxylated species, respectively, commonly observed in oxide-based semiconductors and potentially relevant for interfacial reactions. No evidence of anomalous oxidation states or surface phase segregation was detected.

Quantitative surface compositions coming from XPS (Table S.2) are close to the nominal BiOX stoichiometry within the intrinsic sensitivity limits of the techniques, indicating homogeneous surface chemistry for both materials.

For TiO_2_ P25, XPS analysis was not repeated, as its surface chemical states are extensively reported in the literature and consistent with Ti^4+^ in a mixed anatase–rutile system [60], [61].

The point of zero charge (pH_pzc_) was determined to assess possible electrostatic contributions during IBU abatement. BiOCl and BiOBr exhibit pH_pzc_ values of *ca.* 4.46 and 5.02, respectively (Fig. S4a,b and Table 1), while TiO_2_ P25 shows a value of about 6.5 (Table 1) [50]. Considering the pKa of IBU (4.85), the molecule is predominantly present in its deprotonated, negatively charged form under the experimental conditions [62]. Accordingly, electrostatic interactions are not expected to strongly favour IBU adsorption on the investigated materials under the selected conditions. This interpretation is consistent with the dark uptake observed experimentally, as discussed in Section 3.3.2.

**Fig. 3:**
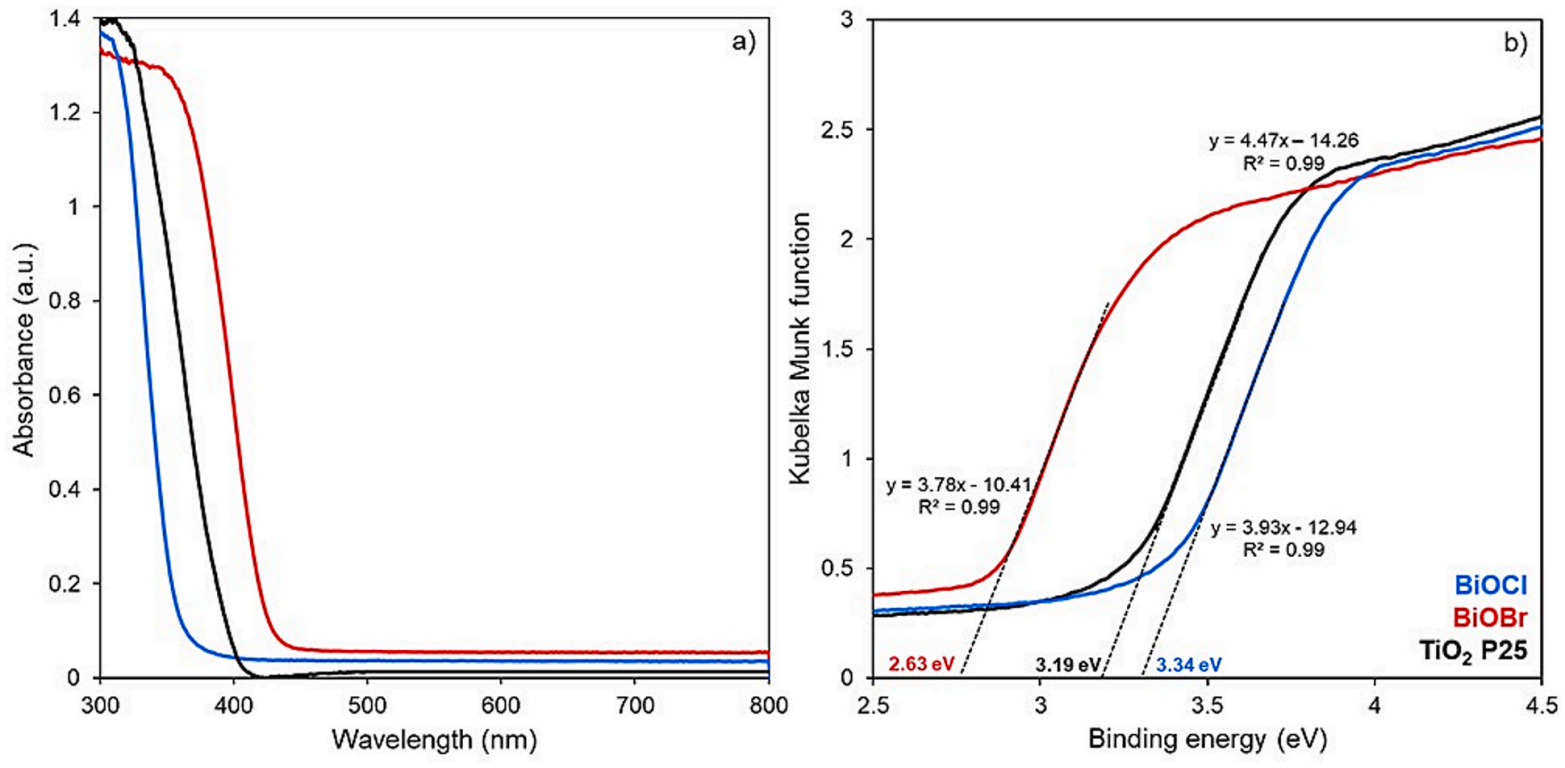
UV–visible absorption spectra (a) and Tauc plots (b) of BiOCl, BiOBr and TiO_2_ P25 powders.

The optical properties of the investigated materials were analysed by UV–DRS (Fig. 3). BiOCl exhibits absorption mainly in the UV region, whereas BiOBr displays a pronounced red-shift with absorption extending into the visible range up to 430 nm. TiO_2_ P25 shows characteristic absorption edge around 380 nm, typical of anatase-based materials [63].

Band gap values derived from Tauc plots (Fig. 3b) are *ca.* 3.34 for BiOCl, 2.63 eV for BiOBr, and 3.19 eV for TiO_2_ P25 (Table 1), in agreement with their respective absorption onsets.

Under the simulated solar irradiation used in this work (35 W·m^−2^), where the visible fraction prevails over the UV component, the broader visible-light absorption of BiOBr is expected to promote more efficient photon utilization.

So, while electrostatic effects remain secondary under the selected operating conditions, the improved visible-light harvesting capability of BiOBr provides a rational basis for its enhanced photocatalytic and sonophotocatalytic performance discussed in the following sections.

**Fig. 4:**
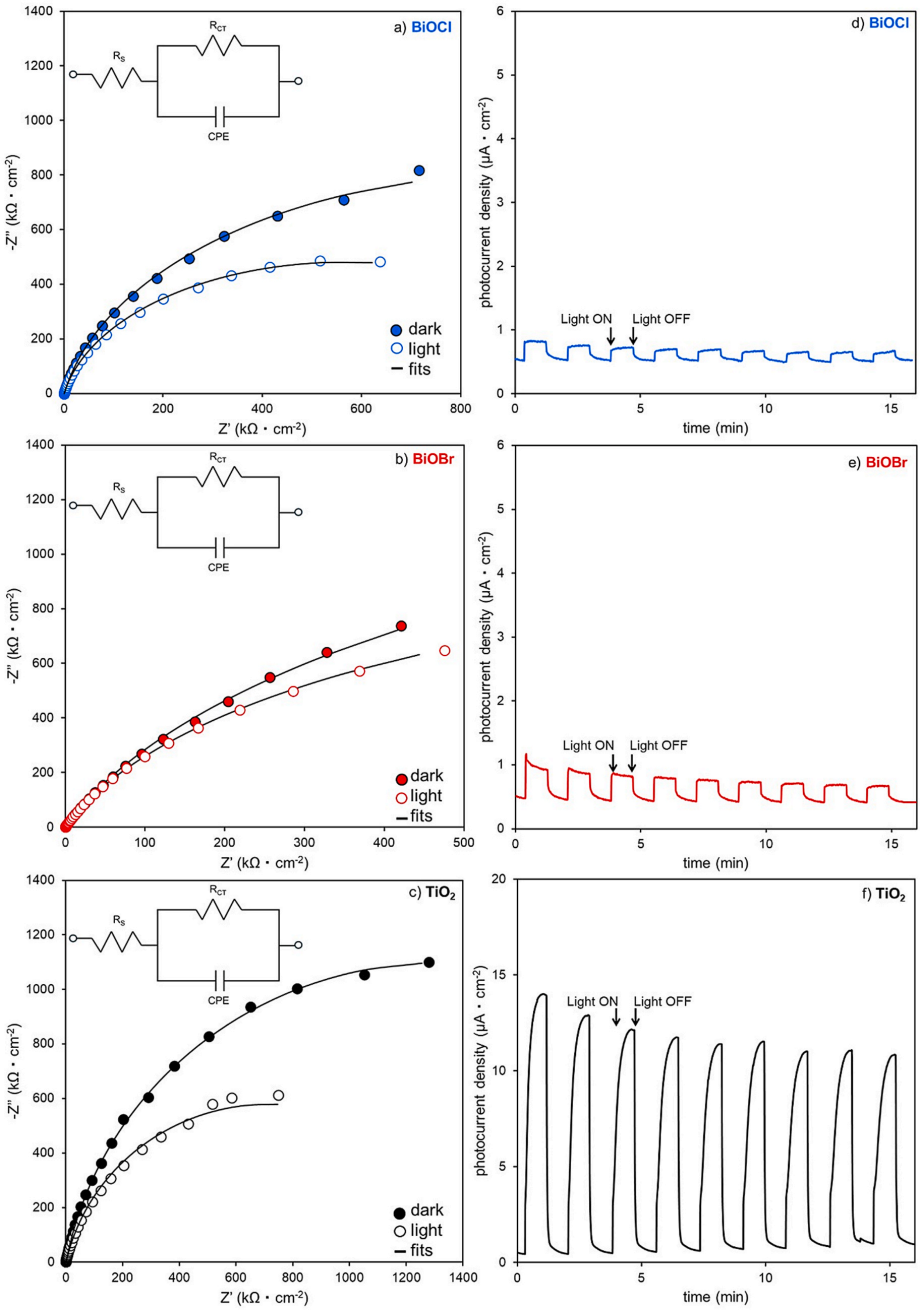
(a-c) Nyquist plots of electrochemical impedance spectroscopy of BiOCl, BiOBr and TiO_2_ P25 under dark (full symbols) and light irradiation (35 W·m^−2^, open symbols); (d-f) Transient photocurrent response of BiOCl, BiOBr and TiO_2_ P25 under dark and light irradiation (35 W·m^−2^).

Electrochemical impedance spectroscopy (EIS) was performed to evaluate the interfacial charge-transfer behaviour of BiOCl, BiOBr and TiO_2_ photoelectrodes under dark and under simulated solar irradiation. The Nyquist plots (Fig. 4a-c) were fitted using a simple RS-(R_CT_||CPE) equivalent circuit, appropriate for describing heterogeneous oxide interfaces [64], and the corresponding fitting parameters are reported in Table S.3.

Under dark conditions, BiOCl exhibits the lowest charge-transfer resistance (R_CT_), followed by TiO_2_ and BiOBr. Upon illumination, all materials show a clear reduction in semicircle diameter, indicating enhanced interfacial charge transfer due to photogenerated carriers. The most pronounced decrease is observed for TiO_2_ (≈ 46% reduction), followed by BiOCl (≈ 40%), while BiOBr shows a more moderate decrease (≈ 29%).

The non-ideal capacitive behaviour observed for all electrodes, reflected in *n* values between 0.85 and 0.90 (Table S.3), is consistent with the heterogeneous nature of oxide-based photoelectrode films. An increase in the CPE magnitude under illumination further supports enhanced charge accumulation at the electrode–electrolyte interface.

These trends are consistent with photocurrent measurements recorded under chopped-light irradiation (Fig. 4d,f). TiO_2_ exhibits the highest photocurrent density (13 µA·cm^−2^) and the most pronounced on–off response, indicating efficient generation and transport of photogenerated charge carriers. BiOBr and BiOCl show lower photocurrent densities (0.75 and 0.34 µA·cm^−2^, respectively), in agreement with their comparatively weaker photoelectrochemical response.

Importantly, although TiO_2_ displays the strongest light-induced decrease in R_CT_ and the highest photocurrent density, this behaviour does not translate into the highest sonophotocatalytic synergy observed in this work. This discrepancy suggests that interfacial photoelectrochemical performance alone is not sufficient to explain the trends under high-frequency cavitation, highlighting the relevance of reactor-imposed effects in the coupled sonophotocatalytic system.

To gain insight into the electronic structure of the studied BiOCl and BiOBr, valence band X-ray photoelectron spectroscopy (VB-XPS) measurements were performed (Fig. S.4c,d).

The valence band maxima (E_VB_), determined by linear extrapolating of the VB edge, were + 2.31 V *vs*. RHE for BiOCl and + 2.19 V *vs*. RHE for BiOBr (Table 1 and Fig. S.4c,d).

By combining these values with the optical band gaps obtained from UV-DRS (Table 1), the corresponding conduction band positions (E_CB_) were estimated to be − 1.03 V *vs.* RHE for BiOCl and − 0.44 V *vs.* RHE for BiOBr. Both materials exhibit sufficiently positive valence band potentials to sustain oxidative pathways under illumination. The more negative conduction band of BiOCl suggests a stronger thermodynamic driving force for reductive processes, whereas BiOBr benefits from a narrower band gap, enabling improved visible-light absorption.

For comparison, the valence band position of TiO_2_ P25 was taken from literature (E_VB_ of *ca.* 2.90 V *vs.* RHE) [65]. Combined with its band gap (3.19 eV, Table 1), this yields an estimated conduction band position of *ca.* −0.20 V *vs.* RHE. While TiO_2_ P25 possesses suitable redox potentials [65], its wide band gap limits effective photon utilization under the simulated solar irradiation used in this work.

**Fig. 5:**
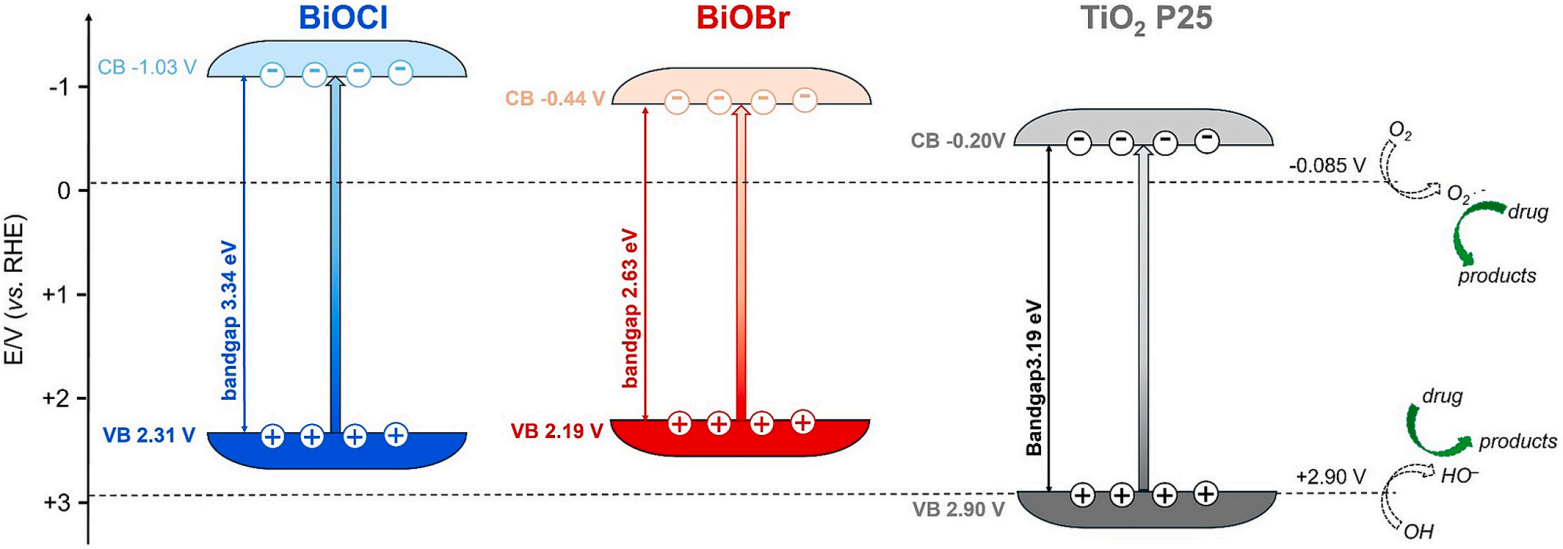
Scheme of band alignments based on VB-XPS and UV–DRS. Data related to TiO_2_ P25 comes from Ref. [65].

A schematic representation of the band alignment and the possible reactive species involved under illumination is presented in Fig. 5. It should be noted that the band-edge position provides thermodynamic indications only; the dominant reactive species involved in the sonophotocatalytic conditions were further investigated through radical scavenging experiments, discussed in Section 3.4.

### 3.2 High-frequency cavitation regime in the plate-type ultrasonic reactor

In ultrasonic-assisted processes, a preliminary characterization of the cavitation regime is essential to correctly analyse catalytic performance. In sonophotocatalytic systems, reactor design and operating frequency directly affect bubble dynamics, spatial distribution of cavitation phenomena, and the balance between mechanical and chemical effects, finally governing radical generation and reaction pathways [18], [66].

Compared to conventional low-frequency horn reactors, high-frequency plate-type systems typically promote a spatially distributed cavitation field in which chemical effects can become predominant [7]. Under these conditions, cavitation effects are mainly chemical, favoring homogeneous radical production rather than localized mechanical erosion. However, the resulting radical yield remains dependent on the balance between bubble population, collapse intensity, acoustic attenuation, and reactor-specific field distribution. These features are particularly advantageous for aqueous-phase advanced oxidation processes, where controlled and reproducible radical generation is required.

To quantitatively evaluate cavitation efficiency and to rationally select the operating conditions for the successive catalytic experiments, potassium iodide (KI) dosimetry was employed as a chemical probe for ·OH formation.

#### 3.2.1 KI dosimetry

KI dosimetry experiments were carried out to compare radical generation efficiency as a function of ultrasonic frequency and operating mode. Tests were performed at 584, 864, and 1148 kHz, applying ultrasound either in continuous mode or in pulsed mode (3 s on / 2 s off), in the absence of catalysts and target pollutants.

**Fig. 6:**
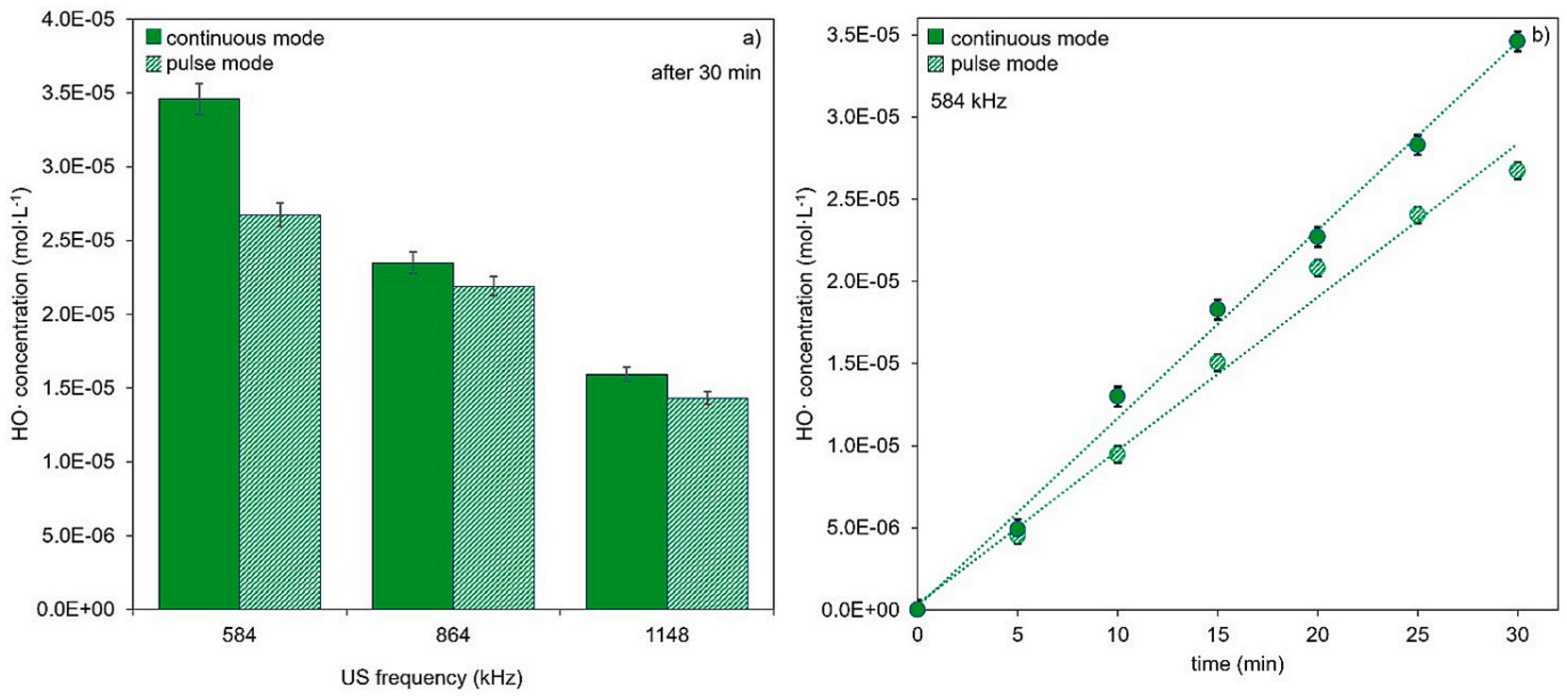
(a) Effect of US frequency on the HO· concentration after 30 min of ultrasonic irradiation in continuous and pulsed mode. (b) Time-dependent evolution of HO· concentration at the selected frequency of 584 kHz. Experimental conditions: [KI] = 0.1 M under dark, US amplitude = 50%.

For all investigated frequencies, applying US in continuous mode resulted in higher HO· concentrations compared to pulsed operation, indicating that sustained acoustic input promotes more efficient radical accumulation under the investigated conditions (Fig. 6 and S.5a,b). Among the tested frequencies, 584 kHz clearly provided the highest radical production, irrespective of the operating mode (Fig. 6a). The time-dependent profile at 584 kHz (Fig. 6b) shows a linear increase of HO· concentration over the investigated time window, consistent with a stable cavitation regime and a constant radical generation rate.

Increasing the ultrasonic frequency to 864 and 1148 kHz led to progressively lower HO· formation.

This behaviour cannot be explained solely by a reduction in bubble size [67]. In sonochemical systems, the radical yield reflects the balance between the number of cavitation events and the chemical effectiveness of each individual collapse [7], [10], [12], [16], [39], [68]. At higher frequencies, the shorter acoustic period limits bubble growth during the rarefaction phase. Although this condition may increase the number density of oscillating bubbles, the reduced expansion ratio can decrease the energy stored within each bubble before collapse. Consequently, individual collapse events may become less chemically effective, with lower local temperatures and pressures and reduced water-vapour dissociation into HO· radicals.

In addition, a larger population of active bubbles can enhance acoustic attenuation and bubble–bubble interactions, reducing the acoustic energy effectively available to individual bubbles. Frequency-dependent changes in the spatial distribution of the acoustic field and in the coupling between the vibrating plate and the liquid volume may further contribute to the observed decrease, particularly in the present reactor geometry. Therefore, the marked decline observed at 1148 kHz is likely associated with a less favourable balance between cavitation-event density and collapse intensity, rather than with bubble size alone. Since direct measurements of bubble populations or local acoustic pressure were not performed, this interpretation is proposed as a physically consistent explanation of the KI-dosimetry trends.

Additional experiments performed in simulated drinking water and under hydrodynamic conditions (Fig. S.5c,d) confirmed that radical generation trends are primarily governed by ultrasonic parameters rather than by matrix effects.

Overall, these results demonstrate that cavitation-driven radical production in the studied plate-type reactor is strongly frequency-dependent. Under the investigated operating conditions and reactor geometry, 584 kHz in continuous mode provided the highest and most stable KI response and was therefore selected for the subsequent sonophotocatalytic experiments.

### 3.3 Ibuprofen (IBU) abatement under high-frequency sonophotocatalytic conditions

Following the identification of the optimal cavitation regime (584 kHz, continuous mode), IBU degradation experiments were carried out to evaluate the relation between high-frequency ultrasound and photocatalysis under controlled conditions.

#### 3.3.1 Non-catalytic baseline processes

IBU abatement was first investigated under non-catalytic conditions to establish a reference framework for rationalizing the catalytic results. Photolysis, sonolysis, and sonophotolysis were performed in ultrapure water (UW) and simulated drinking water (DW) under the same cavitation conditions.

In UW, photolysis led to negligible IBU removal (≈ 8% after 180 min, Fig. S.6a), confirming the limited direct photochemical reactivity of IBU under the applied irradiation conditions. In contrast, sonolysis achieved *ca.* 56% IBU removal (Fig. S.6b), highlighting the key role of cavitation-driven formation of reactive oxygen species (ROS) in pollutant oxidation. The combination of ultrasound and light in the absence of catalyst (sonophotolysis) further increased degradation to about 70% (Fig. S.6c), indicating that dual irradiation enhances the oxidative environment even without a solid surface.

No substantial differences in IBU degradation trends were observed when comparing UW and DW for any of the investigated non-catalytic processes (Fig. S.6, dotted profiles). This evidence is consistent with KI dosimetry results, which showed that radical generation in the investigated high-frequency regime is largely insensitive to dissolved inorganic species (Fig. S.6d). These findings confirm that cavitation-driven oxidation under 584 kHz continuous operation is robust toward matrix composition.

Within the detection limits of HPLC–UV analysis, no clearly resolved transformation products were identified under non-catalytic conditions, suggesting that intermediate species, if formed, remain at low concentration.

#### 3.3.2 Catalytic processes: BiOX *vs*. TiO_2_ P25

**Fig. 7:**
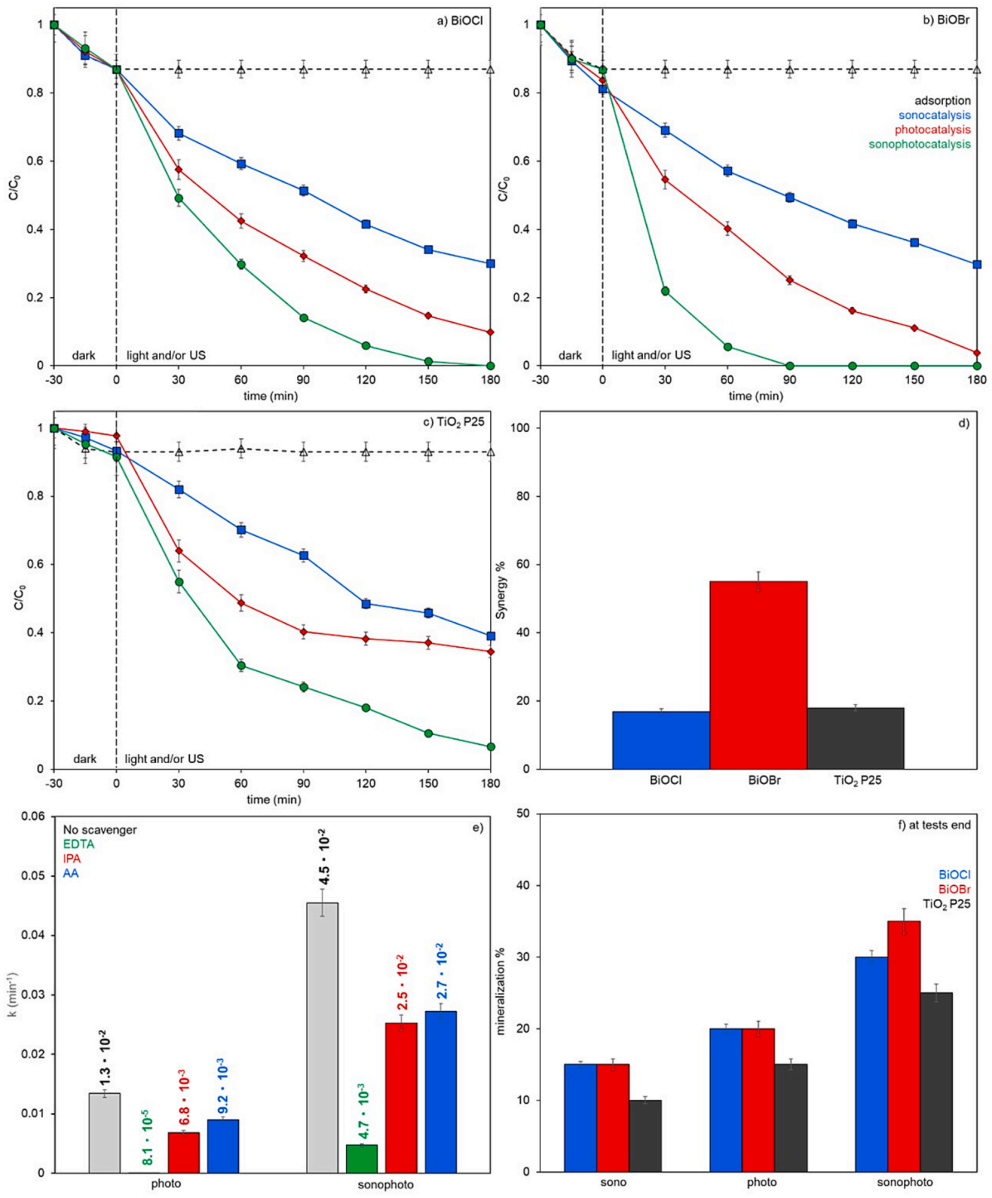
(a-c) IBU abatement in UW on BiOCl, BiOBr and TiO_2_ P25 by adsorption, sono-, photo- and sonophoto-degradation. (d) synergy percentage values for BiOCl, BiOBr and TiO_2_ P25. (e) reactive species trapping results (scavenger-to-IBU molar ratio of 450:1). (f) mineralization percentages at the tests end. Experimental conditions: IBU concentration = 50 mg·L^−1^; catalyst dosage = 0.5 g·L^−1^; irradiance (when used) = 35 W·m^−2^, US continuous mode at 584 kHz, amplitude = 50%, when employed.

The influence of photocatalysts was then investigated using BiOCl, BiOBr and TiO_2_ P25 under photocatalytic, sonocatalytic, and sonophotocatalytic conditions. All experiments were carried out under the optimized cavitation regime identified *via* KI dosimetry (584 kHz, continuous mode), using an initial IBU concentration of 50 mg·L^−1^ and a catalyst dose of 0.5 g·L^−1^. Fig. 7 reports the comparative degradation profiles obtained, while Fig. S.7 and Table S.4 summarize the corresponding kinetic constants and synergy parameters computed.

Before each irradiation experiment, the catalyst suspension was kept in the dark for 30 min under magnetic stirring to allow adsorption–desorption equilibrium to be reached. The residual IBU concentration measured at the end of this equilibration step was then used as the initial concentration (C_0_) for the kinetic analysis. As shown by the dark adsorption profiles (Fig. 7a–c, dotted lines), IBU uptake was below 20% for all catalysts. Therefore, although adsorption was not negligible, the concentration decrease observed after the onset of the selected treatment can be attributed to degradation rather than to continuing surface accumulation. The relatively limited adsorption is consistent with the pH_pzc_ values of the investigated materials (Table 1) and with the predominantly deprotonated form of IBU under the experimental conditions.

Under photocatalytic irradiation, BiOBr exhibited the highest degradation rate, followed by BiOCl, while TiO_2_ P25 showed the lowest activity (Fig. 7a-c). This trend reflects the optical properties previously discussed: the narrower band gap and extended visible-light absorption of BiOBr enable more efficient photon utilization under simulated solar irradiation (35 W·m^−2^), where the visible fraction largely prevails. Conversely, the limited performance of TiO_2_ P25 and BiOCl can be attributed to their preferential activation in the UV region, which represents only a minor fraction of the applied light spectrum [69].

When ultrasound was applied in the absence of light (sonocatalysis), no significant enhancement compared to sonolysis was observed for any of the investigated materials (Fig. 7a-c and S.6b). This indicates that, under high-frequency irradiation, cavitation-induced radical formation in the bulk solution governs the degradation process, while the presence of a solid surface does not substantially alter the purely sonochemical pathway.

A different scenario emerged under simultaneous ultrasound and light irradiation. While BiOCl and TiO_2_ P25 displayed only moderate improvements relative to photocatalysis alone, BiOBr showed a pronounced acceleration of degradation kinetics, reaching near-complete removal within shorter reaction times (Fig. 7b).

This behavior is quantitatively reflected in the kinetic analysis. Pseudo-first-order fits (Fig. S.7) yielded correlation coefficients R^2^ > 0.98 in all cases, confirming the robustness of the applied model. The apparent rate constants and calculated synergy parameters are summarized in Table S.4. For BiOBr, the sonophotocatalytic rate constant (4.55·10^-2^ min^−1^) clearly exceeded the sum of the individual photocatalytic and sonocatalytic contributions (2.04·10^-2^ min^−1^), resulting in a synergy factor of 2.23, corresponding to ≈ 55% synergy.

In contrast, BiOCl and TiO_2_ P25 exhibited synergy factors close to unity (≈ 1.2), indicating essentially additive behaviour under the investigated conditions.

These results demonstrate that synergy in high-frequency plate-type reactors is not an intrinsic consequence of combining ultrasound and light, but depends on how the photocatalyst interacts with the imposed cavitation environment. For BiOBr, the narrower band gap and extended visible-light absorption promote efficient carrier generation under simulated solar irradiation, while high-frequency cavitation supplies a continuous radical flux and may enhance liquid-phase transport near the catalyst surface. Its open flower-like nanosheet assembly may further facilitate access of the reacting solution to exposed and inter-sheet surface regions, allowing the photocatalytic and cavitation-derived pathways to interact more effectively. By comparison, the more compact BiOCl aggregates may be less responsive to this transport contribution, while TiO_2_ P25, despite its higher surface area and favourable photoelectrochemical response, is limited by its weaker activation under the visible-rich irradiation spectrum. Since the selected 584 kHz plate-type configuration is characterized by predominantly chemical cavitation effects and limited mechanical erosion, the observed behaviour is not attributed to substantial ultrasound-induced particle fragmentation or surface restructuring. The results suggest that genuine synergy emerges when visible-light-driven surface reactions, morphology-dependent interfacial accessibility, and the radical-rich environment generated by high-frequency cavitation can operate cooperatively.

Based on its superior kinetics performance and clear synergistic response (Fig. 7d and Table S.4), BiOBr was selected for further investigations.

After use, the structural and textural stability of BiOBr after sonophotocatalysis was assessed (Fig. S.8). The XRD pattern of the used material agreed with the reference BiOBr phase (JCPDS 01–085-0862), with no evidence of phase transformation or loss of crystallinity. N_2_ adsorption–desorption isotherms confirmed that the specific surface area remained comparable to that of the fresh material. These observations indicate that the kinetic enhancement arises from a synergistic interaction between high-frequency cavitation and photocatalysis, rather than from catalyst activation or structural alteration.

#### 3.3.3 Process robustness and operating parameters

To assess the robustness of the process under different operating conditions, the influence of *i)* water matrix composition and *ii)* catalyst dose was investigated, while keeping the initial IBU concentration fixed at 50 mg·L^−1^. This concentration was selected as a representative condition to evaluate the stability of the coupled process.

The effect of water matrix composition was evaluated by comparing the degradation of IBU in UW and DW using BiOBr at a dosage of 0.5 g·L^−1^. The complete concentration profiles obtained in DW under adsorption, photocatalysis, sonocatalysis and sonophotocatalysis are reported in Fig. S.9a, while Fig. S.9b compares the final removal efficiencies in DW with those obtained in UW (Fig. 7b). As expected, the presence of dissolved inorganic species in DW led to a moderate decrease in photocatalytic performance, which can be attributed to a combination of radical scavenging effects (*e.g*., bicarbonate species) and partial attenuation of light reaching the catalyst surface. Nevertheless, sonophotocatalysis maintained a higher efficiency compared to the individual photo- and sono-driven processes, confirming the beneficial role of process coupling also in a more complex aqueous matrix. A closer inspection on kinetics based on a pseudo-first-order model (Table S.5) revealed that the synergistic contribution of ultrasound and light irradiation was largely preserved when moving from UW to DW. Although a decrease in the overall reaction rates was observed, the synergy percentage in DW remained of *ca.* 44%, only slightly lower than that obtained in UW (≈ 55%).

This result clearly indicates that the synergistic behavior of the system is not limited to ideal laboratory conditions, but is retained in the presence of inorganic ions, in agreement with the trends observed by KI dosimetry.

The robustness of the process was further investigated by reducing the BiOBr dosage from 0.5 to 0.25 g·L^−1^, maintaining constant all other operating conditions (Fig. S.9c,d). As expected, lowering the catalyst amount resulted in a decrease in photocatalytic activity due to the reduced availability of active sites. However, the sonophotocatalytic process remained effective, achieving complete IBU removal, even if the kinetics was slower (Fig. S.9c). This behaviour suggests a transition toward a more reaction-controlled regime, where the intrinsic coupling between ultrasound and light irradiation continues to play a dominant role despite the lower catalyst loading.

Also in this case, kinetic investigations (Table S.5) showed that the synergistic effect between ultrasound and light irradiation was still clearly present at 0.25 g·L^−1^ BiOBr, with a synergy percentage of *ca.* 49%. This demonstrates that the combined process does not rely exclusively on high catalyst concentrations, but can operate efficiently even under reduced material usage, an aspect of relevance for process intensification and scale-up considerations.

So, while changes in water matrix composition and catalyst dose affect the absolute degradation rates, the synergistic interaction between ultrasound and light irradiation remains reproducible and robust across the investigated operating conditions.

### 3.4 Reactive species and mechanistic insights

The identification of the dominant reactive species is essential to rationalize the origin of synergy under dual irradiation. Although the band-edge alignment (Fig. 5) provides thermodynamic indications of feasible redox pathways, experimental validation is necessary to determine which species actively govern IBU degradation under the investigated conditions.

Reactive oxygen species (ROS) scavenging experiments were therefore performed using BiOBr as the selected catalyst under photocatalytic, sonocatalytic, and sonophotocatalytic conditions. EDTA, IPA, and AA were used as preferential scavengers for photogenerated holes (h^+^), hydroxyl radicals (·OH), and superoxide radicals (·O_2_^–^), respectively [70]. The corresponding pseudo-first-order kinetic constant**s** are summarized in Fig. 7e and S.10 (R^2^ > 0.98 in all cases).

Under photocatalytic conditions, the addition of EDTA resulted in the strongest inhibition, indicating that photogenerated holes (h^+^) represent the dominant oxidative species (Fig. 7e). The moderate inhibition observed upon addition of IPA and AA suggests that ·OH and ·O_2_^–^ contribute to the degradation process, but do not represent the primary pathway. This trend is consistent with the VB positions derived from VB to XPS (Figs. S.4c,d), which are sufficiently positive to sustain direct hole oxidation.

Under sonocatalytic conditions, the addition of AA led to a pronounced decrease in activity, whereas EDTA and IPA produced negligible effects (Fig. S.10). This result indicates that the degradation pathway inhibited by AA makes the largest apparent contribution to IBU abatement under the investigated high-frequency sonochemical conditions. It should not, however, be interpreted as evidence of a selective intrinsic affinity of IBU for ·O_2_^−^.

KI dosimetry confirms that ·OH is generated during cavitation, but this method quantifies the overall oxidative response of a concentrated iodide solution in the absence of catalyst and pollutant [11], [16], [37], [39]. It does not directly describe the fraction of ·OH that effectively reacts with IBU in the heterogeneous sonocatalytic system. Owing to their extremely short lifetime, ·OH radicals are expected to react close to their generation sites and may be rapidly consumed through competitive reactions, recombination, or conversion into secondary reactive oxygen species before contributing to the measured IBU degradation [71]. Moreover, scavenger tests may not always be sufficiently sensitive to detect the contribution of short-lived ROS when their overall contribution to pollutant degradation is relatively small. Thus, despite the hydroxyl-radical formation evidenced by KI dosimetry, the addition of isopropanol may not produce a measurable variation in the observed degradation kinetics [72], [73], [74].

In the oxygenated catalyst suspension, cavitation-derived radical chemistry and interfacial electron-transfer processes may favour the formation of more persistent oxygen-centred species, including ·O_2_^−^/HO_2_^·^, which may persist and diffuse over longer distances, potentially contributing more effectively to the observed bulk degradation kinetics.

Therefore, the negligible inhibition observed upon IPA addition does not imply the absence of ·OH formation or an inefficient transport of ·OH specifically toward the catalyst surface. It indicates that, under the present conditions, ·OH-mediated oxidation is not the rate-determining pathway captured by the scavenging experiment, whereas the pathway associated with AA-sensitive oxygen-centred species is kinetically more relevant. Since scavenger tests provide indirect mechanistic evidence and may be affected by non-selective reactions, this interpretation is presented as a plausible explanation rather than as direct proof of the individual ROS concentrations or reaction rates.

A distinct and more complex behaviour was observed when combined sonophotocatalytic conditions (Fig. 7e). Strong inhibition in the presence of EDTA confirms the central role of photogenerated holes. Simultaneously, significant reductions in degradation rate upon addition of both IPA and AA indicate ·OH and AA-sensitive oxygen-centred species also contribute to the overall degradation pathway under dual irradiation.

These results support a cooperative multi-pathway mechanism in which surface hole oxidation, cavitation-derived radical reactions, and photoinduced interfacial redox processes operate simultaneously. In addition to generating reactive species in the liquid phase, ultrasound may influence the semiconductor–liquid interface through acoustic microstreaming and local liquid renewal. These effects may facilitate mass transport of dissolved oxygen and reactants to the catalyst surface, reduce the accumulation of reaction products near active sites, and promote the consumption of photogenerated charge carriers through interfacial reactions. In this way, ultrasound could indirectly contribute to more efficient charge-carrier separation by limiting surface charge recombination.

This interpretation is consistent with the simultaneous involvement of h^+^, ·OH, and AA-sensitive oxygen-centred species observed under dual irradiation, as well as with the enhanced degradation kinetics obtained for BiOBr. However, direct evidence of ultrasound-assisted charge separation was not obtained in the present work; therefore, this contribution is proposed as a plausible additional mechanism rather than as a demonstrated origin of the observed synergy.

The open nanosheet assembly of BiOBr may additionally favour the exchange of reactants and cavitation-derived species at the solid–liquid interface, potentially amplifying these transport- and interface-related effects.

Thus, the observed synergy for BiOBr is mechanistically consistent with the interplay between its visible-light-driven charge carrier generation, surface-mediated oxidation, possible ultrasound-assisted interfacial charge consumption, and the radical-rich environment imposed by the selected cavitation regime.

### 3.5 Transformation products and cavitation-assisted reaction pathways

Beyond the IBU disappearance, mineralization represents a more stringent indicator of process effectiveness, as partial oxidation may lead to the accumulation of intermediate TPs [75].

For this reason, total organic carbon (TOC) analyses were performed at the end of the abatement experiments (210 min) under photo-, sono- and sonophotocatalytic conditions, using an initial IBU concentration of 50 mg·L^−1^. Fig. 7f summarizes the mineralization degrees obtained.

Under these conditions, TOC removal followed the same qualitative trend observed for IBU degradation, with sonophotocatalysis clearly overcoming the individual processes for all the investigated catalysts. However, quantitative differences emerged among the materials. Under sonophotocatalytic conditions, TiO_2_ P25 achieved *ca.* 25% TOC removal, BiOCl reached *ca.* 30%, whereas BiOBr exhibited the highest mineralization degree, attaining *ca.* 35%. Although complete mineralization was not achieved within the investigated reaction time, the TOC results provide an important qualification of the apparent degradation performance. In particular, the near-complete disappearance of IBU observed for BiOBr under sonophotocatalytic conditions does not correspond to complete conversion of the organic carbon into inorganic products. At 210 min, *ca.* 65% of the initial organic carbon remained in non-mineralized organic species, likely including transformation products and partially oxidized intermediates. Therefore, the process should not be interpreted as a standalone mineralization technology under the investigated conditions.

Nevertheless, sonophotocatalysis consistently achieved higher TOC removal than the individual photo- and sono-driven processes, particularly in the presence of BiOBr. This indicates that the coupled process promotes a deeper oxidation of IBU-derived intermediates rather than only accelerating the disappearance of the parent compound. Photocatalysis systematically led to higher TOC removal than sonocatalysis alone, despite comparable levels of IBU disappearance, suggesting that ultrasound applied in isolation mainly promotes fragmentation and partial oxidation, whereas surface-mediated photocatalytic reactions are more effective in sustaining sequential oxidation steps. Under dual irradiation, high-frequency cavitation likely intensifies these surface-driven pathways by continuously supplying reactive species and improving interfacial transport, thereby favouring further conversion of intermediate products.

The moderate but systematic increase in TOC removal observed under dual irradiation is consistent with the previously discussed kinetic synergy. While IBU disappearance mainly reflects the initial breakdown of the parent molecule, mineralization provides insight into the progressive deepening of the oxidation network. In this context, the higher TOC removal achieved with BiOBr under sonophotocatalytic conditions indicates that the cooperative interaction between visible-light-driven charge carriers and cavitation-induced radicals favours deeper oxidation of IBU-derived intermediates.

Considering the relatively high initial IBU concentration employed (50 mg·L^−1^) and the 210 min treatment time, the observed mineralization levels are consistent with a sequential oxidation regime in which IBU is rapidly transformed into a complex mixture of oxygenated intermediates that require longer residence times or a subsequent treatment step for more complete mineralization. In this sense, the present system is best positioned as a tertiary polishing or pre-oxidation treatment within an integrated treatment train, and not as a standalone process. Its value lies in the rapid abatement of the parent contaminant, the partial reduction of the organic carbon load, and the modification of the transformation-product distribution before downstream biological, adsorption-based, or complementary oxidation steps. The UHPLC–MS/MS and ECOSAR analyses reported below were therefore included to assess whether this incomplete mineralization is accompanied by an unfavourable accumulation of potentially more hazardous intermediates.

Based on its superior kinetic and mineralization performance, the BiOBr-assisted sonophotocatalytic system was therefore selected for further investigation of TPs. Intermediates were identified by UHPLC–MS/MS, and their potential ecotoxicological relevance was evaluated using ECOSAR modelling to clarify how the imposed high-frequency cavitation regime influences degradation pathways and product distribution.

**Fig. 8:**
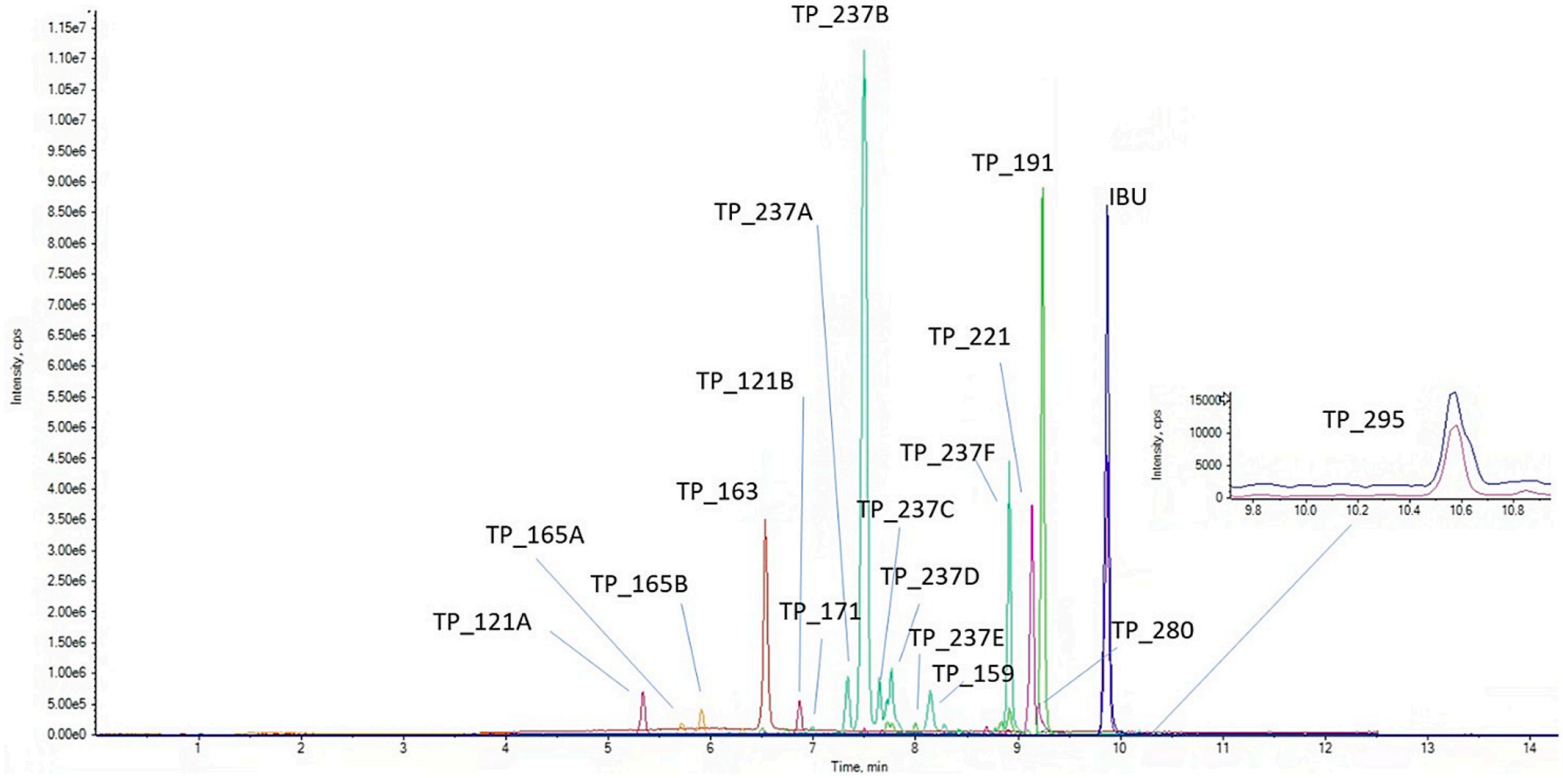
Extracted ion chromatogram of a mixture of sonocatalysis, photocatalysis and sonophotocatalysis experiment solutions at 90 min of degradation, in which the chromatographic peaks of IBU and 17 TPs formed were separated.

For each degradation experiment performed with BiOBr (sonocatalysis, photocatalysis and sonophotocatalysis), the IBU solution at 90 min of degradation time was used to make a mixture to develop an UHPLC-MS/MS method and identify all possible TPs formed. Each TP is associated with the number relating to its *m*/*z* (negative ions), while any letters indicate the presence of isomers. The resulting chromatogram (Fig. 8) shows the good separation of all 17 TPs found, even in the case of isomeric species (TP121, TP165, TP237).

The chemical structures of the unknown TPs formed during degradation were elucidated (Fig. S.11) based on the molecular mass ascertained from the pseudo-molecular ion (M−H^−^), the isotopic pattern obtained in ER mode, the diagnostic fragmentation pathways revealed by tandem mass spectrometry (MS/MS) analysis in EPI scan mode and the reasonableness of the retention time, taking into account the correlation with the calculated log P of TPs. The reactions involved in the formation of TPs are mainly oxidations, hydroxylations on the alkyl chain or aromatic ring, and decarboxylations. Some of the chemical structures of TPs are a confirmation of structures already identified in other studies related to TiO_2_, BiOCl and BiOBr-assisted photocatalytic degradation processes [76], [77], [78], [79], [80], while TP171, TP280, TP295 and some isomers related to TP165, TP121 and TP237 have been identified for the first time and their chemical structures have been proposed. It should be noted that TP237 has 6 dihydroxylated structural isomers on the benzene ring, and since it is not possible to be certain of their position, the structure should be indicated in a generic way with the addition of two OH groups, however, attempts have been made to hypothesize the structures based on both the formation abundance and retention time.

**Fig. 9:**
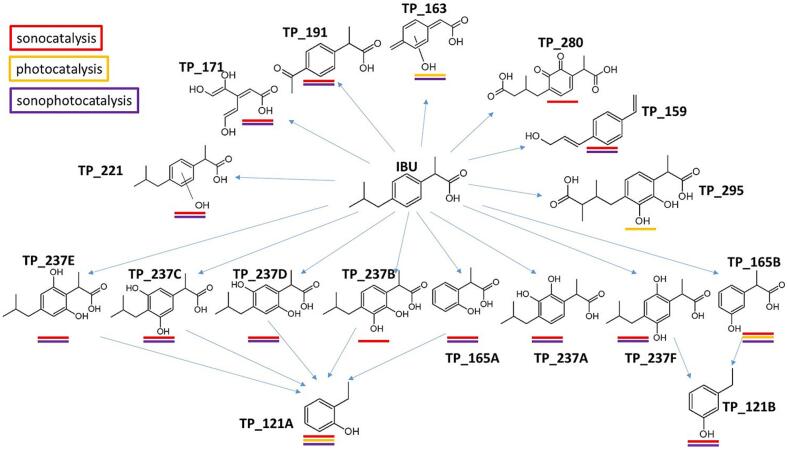
Overall degradation pathway of IBU. The TPs formed in the three different experiments, BiOBr-assisted, are reported: sonocatalysis (red), photocatalysis (yellow) and sonophotocatalysis (purple).

The photocatalysis process generates only 4 TPs, while sonocatalysis and sonophotocatalysis have several TPs in common and generate 15 and 13 TPs, respectively. The progress of each TP was followed by UHPLC-MS/MS, plotting the chromatographic peak area as a function of degradation time (Fig. S.12). The proposed chemical structures of the identified TPs are presented in Fig. 9.

To investigate the potential ecotoxicity of the identified TPs compared to that of the IBU, *in silico* tests were conducted using the ECOSAR software. The following parameters were considered: acute toxicity to freshwater fish (TAF, LC_50_-96 h) and saltwater fish (TASW), daphnid (TAD, LC_50_-48 h), mysids (TAM, LC_50_-96 h), and green alga (TAA, EC_50_-96 h), chronic toxicity to freshwater fish (TCF) and saltwater fish (TCSW), daphnid (TCD), green alga (TCA), and mysids (TCM) in saltwater, bioconcentration factor (BCF), bioaccumulation factor (BAF), and octanol/water partition coefficient (LGP).

Table S.6 reports the predicted endpoint values investigated using Principal Component Analysis (PCA) (Fig. S.13).

The first two principal components explain 74% of the overall variance and show that the variables with the greatest weigh in negative PC1 values are the concentration factor, the bioaccumulation factor, and the octanol/water partition coefficient, while at positive PC1 values are the variables that contribute to acute and chronic toxicity values. PC2, on the other hand, separates tests performed on seawater and tests on mysids (positive PC2 values) from all other tests (negative PC2 values). In the scoring plot, it can be seen that TP171, TP280, and TP191 are significantly less toxic than the predicted toxicity values for IBU, while TP165A, TP165B, and TP295 remain less toxic. However, all other TPs form a cluster with negative PC1 values together with IBU, therefore their predicted toxicity is very similar to that of the IBU precursor. In addition, it can be said that the TPs belonging to this cluster with negative PC1 values also have the same persistence characteristics in water as the IBU: the contribution of bioconcentration factor, the bioaccumulation factor and octanol/water partition coefficient are very similar (negative PC1 in the loading plot).

### 3.6 Process metrics and preliminary economic considerations

While degradation kinetics and mineralization efficiency provide essential information on treatment performance, they do not fully capture the practical feasibility of advanced oxidation technologies [45]. From an engineering perspective, laboratory-scale results should be complemented by process-oriented metrics that account for both energy demand and operational costs [10], [41], [45], [81].

A preliminary assessment was therefore carried out for the high-frequency plate-type ultrasonic reactor operated under the optimized conditions identified in this study (584 kHz, continuous mode, BiOBr-assisted sonophotocatalysis, catalyst dosage of 0.5 g·L^−1^, and initial IBU concentration of 50 mg·L^−1^). The purpose of this analysis was not to provide a full techno-economic assessment, but to contextualize the experimental results within a realistic operational framework.

The cost of ownership (COO) was adopted as an economic figure of merit integrating annualized capital expenditure (CAPEX) and operating expenditure (OPEX), normalized per unit volume of treated water. The assumptions used for the calculation are reported in Table S.7, while the resulting process metrics are summarized in Table 2. A reference treatment capacity of 1 m^3^·h^−1^ operating for 6000 h *per* year was assumed. Capital costs included the ultrasonic reactor, generator, irradiation system, cooling unit, and auxiliary components, and were annualized using a capital recovery factor (CRF) of 0.251, assuming a 5-year equipment lifetime and an 8% discount rate. Operating costs accounted for electricity consumption, routine maintenance, and catalyst make-up, assuming a conservative annual catalyst loss of 5%.

**Table 2:** Representative process metrics for the sonophotocatalytic system investigated in this study.

Parameter	Value	Details
reactor configuration	Plate-type, high-frequency (584 kHz, continuous mode, 50% amplitude)	see Paragraph 2.3.1
process	Sonophotocatalysis in the presence of BiOBr	0.5 g·L^−1^; IBU 50 mg·L^−1^
estimated cost of ownership (COO)	2.20 €·m^−3^	CAPEX + OPEX, according to assumptions reported in Table S.7.
IBU removal target used for EEO	90%	−
EEO for IBU abatement	0.49 kWh·m^−3^·order^-1^	calculated at 90% IBU abatement: it does not represent the energy requirement for complete TOC removal.

Under these assumptions, the estimated COO was *ca.* 2.20 €·m^−3^ (Tables 2 and S.7). This value falls within the range reported for representative cavitation-based treatment technologies, including horn-type ultrasound, flow-through ultrasonic reactors, and hydrodynamic cavitation systems (Fig. S.14) [10]. This comparison should be interpreted cautiously, as the reported values are influenced by differences in reactor scale, treatment objective, operating conditions, and cost boundaries. Nevertheless, it provides a preliminary indication that the proposed high-frequency plate-type configuration does not fall outside the economic range generally associated with cavitation-based water-treatment technologies. This COO value should be interpreted as the estimated cost *per* unit volume processed under the selected operating conditions, and not as the cost required to achieve complete mineralization. Indeed, although BiOBr-assisted sonophotocatalysis led to rapid IBU removal, TOC removal reached approximately 35% after 210 min. Therefore, the process promotes partial oxidation and transformation of the organic load rather than complete conversion of organic carbon into inorganic products within the investigated treatment time.

To relate electrical demand to the observed abatement performance, the electrical energy *per* order (EEO) was calculated for IBU removal. Under the optimized BiOBr-assisted sonophotocatalytic conditions, an EEO of 0.49 kWh·m^−3^·order^−1^ was obtained for 90% IBU abatement (Table 2). This value represents the electrical energy required to reduce the concentration of the parent compound by one order of magnitude and provides a performance-normalized estimate of the energy demand associated with the combined ultrasound–light process. The calculation includes the total electrical power demand of the ultrasonic reactor, irradiation source, and cooling system. However, because mineralization remained incomplete, the EEO was not extrapolated to full TOC removal and should not be interpreted as the energy requirement for complete mineralization.

The combined interpretation of COO and EEO is important in the present case. EEO captures the electrical energy required to reach a defined IBU abatement target, whereas COO additionally includes capital amortization, maintenance, catalyst make-up, and auxiliary utilities [10], [45]. The observed kinetic synergy should therefore be evaluated not only in terms of faster parent-compound disappearance, but also in relation to the intended treatment objective. Under the present conditions, the additional ultrasonic input is associated with enhanced degradation kinetics and higher mineralization relative to the individual processes. Nevertheless, the moderate TOC removal indicates that the configuration is more appropriately positioned as a tertiary polishing or pre-oxidation step within an integrated treatment train than as a standalone mineralization technology.

Beyond the absolute values of the calculated metrics, the high-frequency plate-type configuration presents relevant practical features. Under high-frequency operation, cavitation effects are predominantly chemical rather than erosive, potentially reducing mechanical wear compared with low-frequency horn systems. The absence of immersed moving parts and the compatibility with low-intensity irradiation may further support operational stability and modular scalability. At the same time, the present estimates remain sensitive to catalyst durability, recovery efficiency, electricity cost, and reactor lifetime, which should be assessed at pilot scale before drawing definitive conclusions on industrial implementation [82].

So, the preliminary COO and EEO values indicate that high-frequency plate-type sonophotocatalysis with BiOBr is a technically plausible intensification strategy for rapid IBU abatement and partial oxidation. More broadly, these findings reinforce the central message of this work: reactor-driven design choices influence not only kinetic performance and synergy, but also energy demand and the practical positioning of sonophotocatalytic processes.

## 4 Conclusions

High-frequency sonophotocatalysis was systematically investigated using a plate-type ultrasonic reactor operating at 584 kHz to elucidate the origin of synergistic effects in combined ultrasound–photocatalytic systems. KI dosimetry demonstrated that radical generation is strongly dependent on the imposed cavitation regime, identifying 584 kHz in continuous mode as the most effective and stable operating condition.

Under these controlled conditions, true synergy was observed only for BiOBr, whose sonophotocatalytic rate constant exceeded the sum of the individual photocatalytic and sonocatalytic contributions by a factor of 2.23 (≈55% synergy). In contrast, BiOCl and TiO_2_ P25 exhibited essentially additive behaviour, demonstrating that the simultaneous application of ultrasound and light does not inherently lead to cooperative effects.

Reactive species trapping experiments revealed that synergy originates from a cooperative multi-pathway mechanism involving photogenerated holes together with cavitation-induced reactive oxygen species. The visible-light response and suitable band alignment of BiOBr enable an efficient coupling between photocatalytic surface reactions and the radical-rich environment generated under high-frequency cavitation. Its open nanosheet morphology may additionally promote interfacial accessibility and local liquid renewal under acoustic irradiation, further supporting this cooperative response. The preservation of the synergistic contribution in simulated drinking water and at reduced catalyst loading further indicates that this reactor–catalyst interplay remains robust beyond the reference conditions.

Although complete mineralization was not achieved within the investigated reaction time, sonophotocatalysis consistently enhanced TOC removal compared with the individual processes. The difference between rapid IBU disappearance and the moderate TOC removal (up to *ca.* 35% after 210 min) confirms that the system promotes sequential oxidation rather than complete mineralization under the tested conditions. Accordingly, high-frequency sonophotocatalysis should be regarded as a tertiary polishing or pre-oxidation step within an integrated treatment train, rather than as a standalone mineralization technology. Its practical role may therefore lie in the rapid abatement of the parent contaminant, partial reduction of the organic load, and modification of the transformation-product distribution before downstream treatment.

A preliminary process-oriented assessment yielded an estimated cost of ownership of ∼ 2.20 €·m^−3^ and an EEO of 0.49 kWh·m^−3^·order^-1^ for 90% IBU abatement. These values should be interpreted in relation to parent-compound removal and partial oxidation, and not as metrics for complete TOC mineralization. The COO estimate falls within the broad range reported for representative cavitation-based treatment technologies, although differences in scale, operating conditions, treatment objectives, and cost boundaries prevent a direct economic comparison.

The present study demonstrates that synergy in sonophotocatalysis is not an intrinsic consequence of process coupling, but is critically governed by the cavitation regime imposed by the reactor configuration together with the electronic structure of the photocatalyst. By linking cavitation characterization, catalyst-dependent kinetic synergy, oxidation depth, and preliminary process metrics, this work provides a reactor-oriented framework for the rational and scale-aware development of high-frequency sonophotocatalytic systems.

## CRediT authorship contribution statement

**Vincenzo Fabbrizio:** Writing – original draft, Visualization, Methodology, Investigation, Formal analysis, Data curation. **Melissa G. Galloni:** Writing – review & editing, Writing – original draft, Visualization, Validation, Methodology, Investigation, Formal analysis, Data curation, Conceptualization. **Ermelinda Falletta:** Visualization, Validation, Formal analysis. **Enrique Rodriguez Castellon:** Formal analysis. **Giuseppina Cerrato:** Visualization, Formal analysis. **Fabio Gosetti:** Writing – review & editing, Writing – original draft, Visualization, Validation, Resources, Methodology, Investigation, Formal analysis, Data curation, Conceptualization. **Claudia L. Bianchi:** Writing – review & editing, Visualization, Validation, Supervision, Resources, Funding acquisition, Conceptualization.

## Funding

Velux Stiftung Foundation is gratefully acknowledged for the financial support through the project 1381 “SUNFLOAT—Water decontamination by sunlight-driven floating photocatalytic systems”.

This work was supported by Dipartimento di Chimica, Università degli Studi di Milano, Italy (Piano Sostegno alla Ricerca, PSR, grant 2022). E. Rodríguez-Castellón thanks Ministry of Science and Innovation of Spain for the grant PID2021-126235OB-C32 financed by MCIN/AEI/501100011033 and the FEDER funds.

## Declaration of competing interest

The authors declare that they have no known competing financial interests or personal relationships that could have appeared to influence the work reported in this paper.
