# Supplementary material for: Cavitation-regime-controlled synergy in high-frequency sonophotocatalysis
Source: Ultrason Sonochem. 2026 Aug 5;132:107996. doi: 10.1016/j.ultsonch.2026.107996 (PMC13476520; doi:10.1016/j.ultsonch.2026.107996)
Supplement: Supplementary file 1 [file mmc1.docx]

**Supporting Material**

**Cavitation-regime-controlled synergy in high-frequency sonophotocatalysis**

Vincenzo Fabbrizio,^1,2^ Melissa G. Galloni,^1,2,*^ Ermelinda Falletta,^1,2^ Enrique Rodriguez Castellon,^3^ Giuseppina Cerrato,^2,4^ Fabio Gosetti,^5^ Claudia L. Bianchi^1,2^

*^1^ Dipartimento di Chimica, Università degli Studi di Milano, via Golgi 19, 20133 Milano, Italy*

*^2^* *Consorzio Interuniversitario Nazionale per la Scienza e Tecnologia dei Materiali (INSTM), Via Giusti 9, 50121 Firenze, Italy*

*^3^ Department of Inorganic Chemistry, Crystallography, and Mineralogy, Faculty of Sciences, Instituto Interuniversitario de Investigación en Biorrefinerías I3B, University of Malaga, 29071 Málaga, Spain*

*^4^ Dipartimento di Chimica, Università degli Studi di Torino, Via Giuria 7, 10125 Torino, Italy*

*^5^ Department of Earth and Environmental Sciences, Università degli Studi Milano-Bicocca, Milano, Italy*

***AUTHORS’ INFORMATION***

*Corresponding Author*

**E-mail: melissa.galloni@unimi.it*

*ORCID*

*Vincenzo Fabbrizio: 0009-0008-4449-5582*

*Melissa Greta Galloni: 0000-0001-5446-5186*

*Ermelinda Falletta: 0000-0002-3127-8967*

*Enrique Rodríguez-Castellón: 0000-0003-4751-1767*

*Giuseppina Cerrato:* *0000-0001-8536-1398*

*Fabio Gosetti:* *0000-0001-5712-0562*

*Claudia L. Bianchi: 0000-0002-9702-6949*

**Materials**

Bismuth(III) nitrate pentahydrate (Bi(NO_3_)_3_·5H_2_O, 98%), potassium bromide (KBr, > 99.5%), potassium chloride (KCl, > 99.5%), and glacial acetic acid (CH_3_COOH, pure) were of analytical grade and supplied by Merck. Commercial titanium dioxide P25 (TiO_2_ P25, > 99.5%) was purchased from Sigma-Aldrich.

For materials’ characterization, liquid nitrogen (N_2_, > 99.0%, Sapio), nitric acid (HNO_3_, 65.0%, Carlo Erba), sodium hydroxide (NaOH, > 99%, Carlo Erba), sodium nitrate (NaNO_3_, > 99%, Merck), barium sulphate (BaSO_4_, > 99.0%, Nacalai Tescue), and sodium sulphate (Na_2_SO_4_, > 99%, Merck) were used.

For abatement tests and the preliminary potassium iodide (KI) dosimetry experiments, potassium iodide (KI, > 99.0%, Sigma-Aldrich), hydrochloric acid (HCl, 37%, Sigma-Aldrich), ibuprofen sodium salt (C_13_H_17_NaO_2_, > 98.0%, Sigma-Aldrich), disodium ethylenediaminetetraacetic acid dihydrate (C_10_H_14_N_2_Na_2_O_8_, > 99.5%, Merck), magnesium sulphate (MgSO_4_, > 99.5%, Sigma-Aldrich), calcium chloride (CaCl_2_, > 96%, Merck), sodium bicarbonate (NaHCO_3_, > 99%, Merck), 2-propanol ((CH_3_)_2_CHOH, > 99.5% Sigma-Aldrich), L-ascorbic acid (C_6_H_8_O_6_, 98%, Sigma-Aldrich), acetonitrile (CH_3_CN, HPLC grade, Carlo Erba), water (H_2_O, HPLC grade, Carlo Erba), formic acid (HCOOH, 98%, Sigma-Aldrich) were employed.

For the UHPLC-MS/MS analysis, water (UHPLC-MS grade), acetonitrile (UHPLC-MS grade) and formic acid (LC-MS grade) were acquired from Carlo Erba (Milan, Italy).

Ultra-pure water was obtained using a Milli-Q® ultrapure water system (Merck Millipore).

**Materials characterisation**

The crystal structure and purity of the investigated materials were analyzed by X-ray powder diffraction (XRD) using a PANanalytical X’Pert PRO diffractometer equipped with a Cu Kα radiation source (λ = 1.54060 Å) provided with an X-ray source operating at 40 kV x 40 mA. Diffractograms were collected in the 2θ range of 10–80° using a step size of 0.02° and counting times between 5 and 96 s *per* step. Phase identification was carried out by comparison with JCPDS reference files from the International Center for Diffraction Data.

Morphology and elemental distribution were examined by field-emission scanning electron microscopy (FESEM) using a TESCAN S9000G microscope (Overcoached, Germany) equipped with a Schottky type FEG source (resolution 0.7 nm at 15 keV in In-Beam SE mode) and an Oxford Ultim Max EDS detector.

For SEM analyses, samples were mounted on Al metallic stabs using conductive carbon tape and coated with a thin chromium layer by ion sputtering to improve surface conductivity.

Specific surface area was determined by N_2_ adsorption-desorption isotherms at -196°C using a Micromeritics Tristar 3Flex analyzer. Prior to N_2_ adsorption-desorption measurements, *ca*. 1 g each sample was dried at 120°C overnight and successively was treated at 150°C for 4 h under nitrogen flow (Air Liquide Alphagaz) to remove physisorbed species from the surface. Specific surface areas were calculated using the Brunauer-Emmet-Teller (BET) equation (2-parameters) in the relative pressure range 0.05 < p/p° < 0.3, assuming a nitrogen cross-sectional area of 16.2 Ǻ.

Surface chemical composition and oxidation states were studied by X-ray photoelectron spectroscopy (XPS) using a PHI Versa-Probe II scanning XPS microprobe (Physical Electronics) spectrometer, and valence band XPS (VB-XPS) measurements were carried out to determine the position of the valence band edge in BiOBr and BiOCl, using the same instrument.

XPS analyses were performed using a monochromatic Al Kα X-ray source (h*ν* = 1486.6 eV) and a charge neutralizer operating at 100 W and 20 kV under a vacuum (~10^-7^ Pa). Spectra were processed using PHI SmartSoft software and MultiPak 9.3 package. Binding energies were calibrated using the adventitious C *1s* peak at 284.8 eV. VB-XPS spectra were recorded with a pass energy of 29.35 eV and an energy step of 0.125 eV to ensure high resolution near the Fermi level. Energy calibration was performed using Cu *2p_3/2_* (932.7 eV), Ag *3d_5/2_* (368.2 eV), and Au *4f_7/2_* (83.95 eV) reference peaks. The valence band maximum values obtained from XPS (E_VB-XPS_) were converted into electrochemical potentials *vs.* the reversible hydrogen electrode (RHE) according to the following equation:

$E \left( V vs. NHE \right)= -\left( E_{VB-XPS}+4.44 \right)$	(eq. S.1)

where 4.44 eV corresponds to the energy difference between the vacuum level and the normal hydrogen electrode (NHE). Subsequently, the conversion from NHE to RHE was carried out according to the following equation:

$E \left( V vs. RHE \right)=E_{NHE}+0.059\cdot pH$	(eq. S.2)

All band edge positions discussed in this work are reported *vs.* RHE.

The point of zero charge (pH_pzc_) was determined by the pH drift method using 0.1 M NaNO_3_ solutions with initial pH values adjusted between 4.0 and 10.0. After 24 h of stirring, suspensions were centrifuged and final pH values were measured. The pH_pzc_ was obtained as the pH at which ΔpH (pH_final_ − pH_initial_) equals zero.

Optical properties were investigated by UV-vis diffuse reflectance spectroscopy (UV-DRS) in the 200-800 nm range using a PerkinElmer Lambda 750s UV-Vis spectrophotometer. UV-DR spectra were collected by grinding and pressing each sample into disks (*ca.* 4 cm diameter), placed at the integrating sphere window of the spectrophotometer. BaSO_4_ was used as the reflectance standard. Data were processed to obtain absorbance versus wavelength plots, followed by Tauc plots for the determination of bandgap values.

Electrochemical measurements were performed in a three-electrode glass cell (total volume 100 mL) connected to an Autolab PGSTAT204 potentiostat. Samples-modified indium tin oxide (ITO) electrodes were prepared by dispersing 10 mg catalyst in 400 μL of acetone under sonication, followed by drop-casting 20 μL suspension onto ITO glass substrates (50 × 50 mm^2^) and drying overnight at room temperature. Measurements were carried out in 0.1 M Na_2_SO_4_ aqueous solution after purging with N_2_ for 30 min to remove dissolved oxygen. Simulated solar irradiation was provided by an OSRAM Ultra-Vitalux 300 W lamp (35 W·m^-2^).

Electrochemical Impedance Spectroscopy (EIS), and transient photocurrent measurements were performed in a single-compartment three-electrode glass cell to investigate charge transport, band structure, and photoinduced charge-carrier dynamics. The investigated materials were used as working electrodes, a saturated calomel electrode (SCE) as reference, and a platinum counter electrode, with all measurements carried out using a Metrohm Autolab PGSTAT204 potentiostat/galvanostat. All electrochemical potentials were converted to the reversible hydrogen electrode (RHE) scale for comparison.

Electrochemical impedance spectroscopy (EIS) measurements were performed under potentiostatic conditions at +1.55 V *vs.* RHE, both in the dark and under simulated solar irradiation (35 W·m^-2^). Nyquist plots were obtained by plotting the imaginary component of the impedance (−Z”) versus the real component (Z’). The experimental spectra were subsequently fitted using the NOVA 2.1 software (Metrohm Autolab) to extract the relevant electrochemical parameters. The fitting procedure was carried out using the selected equivalent electrical circuit, and the quality of the fitting was assessed based on the χ^2^ values and the agreement between experimental and simulated data.

Transient photocurrent measurements were carried out under chopped light irradiation (50 s on/off cycles) at +1.55 V *vs.* RHE to evaluate photoinduced charge-carrier dynamics. Photocurrent densities were calculated as the difference between the steady-state current under illumination and that recorded in the dark.

Table S.1. Electrolyte contents of the simulated drinking water (DW).

| Species | Concentration (mg·L^-1^) |
| --- | --- |
| Ca^2+^ | 44.5 |
| Na^+^ | 45.7 |
| Mg^2+^ | 9.3 |
| Cl^-^ | 78.7 |
| SO_4_^2-^ | 36.5 |
| HCO_3_^-^ | 121.2 |

**Ibuprofen abatement tests**

Ibuprofen (IBU) abatement experiments were performed using 300 mL of aqueous IBU solution (10 or 50 mg·L^-1^) prepared in water (ultrapure, UW or simulated drinking water, DW) at spontaneous pH. When used, the catalyst dosage was fixed at 0.25 or 0.5 g·L^-1^. Prior to irradiation, suspensions were kept in the dark for 30 min to reach adsorption–desorption equilibrium; the concentration measured after this step was used as the initial concentration (C_0_) for kinetic calculations. Due to the absence of mechanical stirring in the plate-type ultrasonic reactor, this pre-adsorption step was carried out under magnetic stirring (250 rpm) in a separate glass vessel. The suspension was then transferred to the ultrasonic reactor and immediately subjected to the selected irradiation conditions.

During the experiments, aliquots (1.5 mL) were sampled at selected time intervals, filtered through 0.22 μm nylon syringe filters, and transferred into 2 mL vials for quantitative analyses.

Four operating conditions were systematically investigated: photolysis (light only), sonolysis (ultrasound only), sonophotolysis (simultaneous ultrasound and light in the absence of catalyst), and catalytic processes (photocatalysis, sonocatalysis, and sonophotocatalysis, in the presence of TiO_2_ P25, or BiOCl or BiOBr).

The variation of IBU concentration was monitored by HPLC–UV using an Agilent 1100 Series instrument equipped with a C18 Sulpelco column (25 cm × 4 mm, 5 μm), a 20 μL injection loop, and a UV-vis detector. Isocratic elution was performed using a water/acetonitrile/formic acid (50/49.9/0.1, v/v/v) mobile phase at 1 mL·min^-1^ flow rate with UV detection at 230 nm for IBU and 260 nm for transformation products (qualitative evaluation).

In this way, profiles of C/C_0_ as a function of time, where *C* is the pollutant concentration at the time *t* and *C_0_* is the initial concentration of the target pollutant along with time in minutes, were obtained.

IBU abatement was computed by the equation:

$Pollutant abatement \left( \% \right)= \frac{C_{0}-C}{C_{0}} \cdot100\%$	(eq. S.3)

Mineralization was evaluated by total organic carbon (TOC) analyses using a Shimadzu TOC-L analyzer when an initial IBU concentration of 50 mg·L^-1^ was employed. TOC samples were collected from the initial solution and at the end of the experiments (210 min), and mineralization efficiency was calculated according to the following equation:

$Mineralization capacity \left( \% \right)= \frac{\mathrm{TOC}_{0}- \mathrm{TOC}_{f}}{\mathrm{TO}C_{0}} \cdot100\%$	(eq. S.4)

where TOC_0_ is the initial TOC concentration, and TOC_f_ is the final one at the end of the experiments.

IBU degradation kinetics were evaluated using a pseudo-first-order model, according to the equation:

$\ln\frac{C_{0}}{C}=kt$	(eq. S.5)

where *C_0_* is the IBU concentration measured after the 30 min dark adsorption-desorption equilibration step, *C* is the pollutant concentration at reaction time *t*, *k* is the kinetic constant, and *t* is the reaction time. In all cases, fits with R^2^ values higher than 0.98 were detected.

The synergy factor and the percentage of synergy were calculated by comparing the rate constant of the sonophotocatalytic process with the sum of the individual photocatalytic and sonocatalytic contributions as follows.

$\mathrm{Synergy} \mathrm{factor} = \frac{k \mathrm{sonop}h\mathrm{otocatalysis}}{k \mathrm{sonocatalysis} + k ph\mathrm{otolcatalysis}}$	(eq.S.6)

% 𝑠𝑦𝑛𝑒𝑟𝑔𝑦 = $\frac{k \mathrm{sonop}h\mathrm{otocatalysis} - (k sonocatalysis + k ph\mathrm{otocatalysis})}{k \mathrm{sonophotocatalysis}}$ · 100%	(eq.S.7)

Reactive species trapping experiments were carried out to investigate the role of photogenerated holes (h⁺), hydroxyl radicals (·OH), and superoxide radicals (·O_2_⁻) in IBU abatement (50 mg·L^-1^) when BiOBr was used as catalyst (dose = 0.5 g·L^-1^). Disodium ethylenediaminetetraacetic acid (EDTA), isopropyl alcohol (IPA), and ascorbic acid (AA) were used as scavengers for h⁺, ·OH, and ·O_2_⁻, respectively. Scavengers were added after the dark adsorption step at a scavenger-to-IBU molar ratio of 450:1, ensuring an excess of scavenger to effectively inhibit the target reactive species. Successive photocatalytic, sonocatalytic, and sonophotocatalytic experiments were performed under the same operating conditions described above.

All experiments were repeated at least three times to ensure reproducibility with a percent relative uncertainty of less than 5%. Error bars representing the standard deviation of the measurements are included in the figures where applicable.

Figure S.1. FESEM images of BiOCl [1] and BiOBr (a,b) and N_2_ adsorption-desorption isotherms at

-196°C (c-e) of BiOCl, BiOBr and TiO_2_ P25.

Figure S.2. Survey (a) and HR XP spectra of C 1*s* (b), O 1*s* (c), Bi 4*f* (d), and Cl *2p* (e) for BiOCl. Deconvolutions in sub-bands and the resulting fitted spectra (black lines) are also reported.

Figure S.3. Survey (a) and HR XP spectra of C 1*s* (b), O 1*s* (c), Bi 4*f* (d), and Br 3*d* (e) for BiOBr. Deconvolutions in sub-bands and the resulting fitted spectra (black lines) are also reported.

Table S.2. Surface composition by XPS.

| Code | surface composition  (atomic %) | | |
| --- | --- | --- | --- |
|  | C | O | other elements |
| TiO_2_ P25 | 40.5^a^ | 43.2^a^ | Ti: 16.3^a^ |
| BiOCl | 26.6 | 30.3 | Bi: 16.1; Cl: 27 |
| BiOBr | 27.2 | 25.9 | Bi: 28.4; Br: 18.5 |

^a^from Ref. [2].

Figure S.4. Point of zero charge determination (a,b) and VB-XPS spectra (c,d) for BiOCl and BiOBr.

Table S.3. Fitting parameters derived from EIS analysis of BiOCl, BiOBr and TiO_2_ under dark and illuminated conditions (35 W·m^-2^), obtained using the R_S_–(R_CT_∥CPE) equivalent circuit in NOVA 2.1.

| Code | dark | | | | | light | | | | |
| --- | --- | --- | --- | --- | --- | --- | --- | --- | --- | --- |
|  | R_S_ | R_CT_ | Y_0_ | n | χ^2^ | R_S_ | R_CT_ | Y_0_ | n | χ^2^ |
| BiOCl | 0.589 | 1883 | 0.0008 | 0.900 | 0.00033 | 0.533 | 1135 | 0.0009 | 0.895 | 0.00023 |
| BiOBr | 0.220 | 2841 | 0.001 | 0.851 | 0.0023 | 0.217 | 2028 | 0.001 | 0.852 | 0.0022 |
| TiO_2_ | 0.370 | 2627 | 0.0004 | 0.885 | 0.00027 | 0.310 | 1430 | 0.0008 | 0.869 | 0.00028 |

Figure S.5. (a, b) Time-dependent evolution of HO· concentration at different operating frequencies (584, 864, 1148 kHz) applying US in pulse (3 seconds on/ 2 seconds off) or continuous mode. (c, d) Effects of blade stirrer and water matrix on the evolution of HO· concentration along with time applying US at 584 kHz in continuous mode. Experimental conditions: [KI] = 0.1 M under dark.

Figure S.6. Effect of water matrix (UW vs. DW, *i.e*., full vs. empty symbols) on non-catalytic IBU abatement by photolysis, sonolysis and sonophotolysis.

Experimental conditions: [IBU] = 50 mg·L^-1^, irradiance (when used) = 35 W·m^-2^, US continuous mode at 584 kHz, amplitude = 50%, when employed.

Figure S.7. Linear fitting of pseudo-first-order kinetics for IBU abatement in UW on BiOCl, BiOBr and TiO_2_ P25.

Experimental conditions: IBU concentration = 50 mg·L^-1^; catalyst dosage = 0.5 g·L^-1^; irradiance (when used) = 35 W·m^-2^, US continuous mode at 584 kHz, amplitude = 50%, when employed.

Table S.4. Comparison of catalytic results and synergy obtained for BiOX and TiO_2_ P25.

| Code | k_sonocatalysis_^a^ (min^-1^) | k_photocatalysis_^a^ (min^-1^) | k_sonophotocatalysis_^a^ (min^-1^) | k_sonocatalysis_+k_photocatalysis_ (min^-1^) | Synergy factor^b^  (%) | Synergy %^c^ |
| --- | --- | --- | --- | --- | --- | --- |
| BiOCl | 5.9·10^-3^ | 1.15·10^-2^ | 2.09·10^-2^ | 1.74·10^-2^ | 1.20 | 17 |
| BiOBr | 5.5·10^-3^ | 1.49·10^-2^ | 4.55·10^-2^ | 2.04·10^-2^ | 2.23 | 55 |
| TiO_2_ | 4.9·10^-3^ | 7.1·10^-3^ | 1.46·10^-2^ | 1.19·10^-2^ | 1.22 | 18 |

^a^ according to pseudo-first-order kinetics; ^b^ computed by eq. S.6; ^c^ computed by eq. S.7.

Figure S.8. (a) XRD pattern after sonophotocatalytic IBU abatement, compared with the reference diffraction pattern (JCPDS 01-085-0862) and (b) N_2_ adsorption-desorption isotherms at -196°C of used BiOBr.

Figure S.9. Effect of water matrix: (a) IBU abatement in DW; (b) comparative view of IBU abatement in UW *vs.* DW at the tests end. Conditions: [IBU] = 50 mg·L^-1^, catalyst dose = 0.5 g·L^-1^.

Effect of BiOBr dose: (c) IBU abatement by 0.25 g·L^-1^ BiOBr; (d) comparative view of IBU abatement on 0.5 or 0.25 g·L^-1^ BiOBr at the tests end. Conditions: [IBU] = 50 mg·L^-1^.

In all tests: irradiance (when used) = 35 W·m^-2^, US continuous mode at 584 kHz, amplitude = 50%, when employed, whereas adsorption was carried out in the dark.

Table S.5. Effect of operating parameters on IBU abatement by BiOBr.

| Catalyst used | Experimental conditions^a^ | Studied effect | k_sonocatalysis_^b^  (min^-1^) | k_photocatalysis_^b^  (min^-1^) | k_sonophotocatalysis_^b^ (min^-1^) | k_sonocatalysis_+k_photocatalysis_ (min^-1^) | Synergy factor^c^  (%) | Synergy %^d^ |
| --- | --- | --- | --- | --- | --- | --- | --- | --- |
| BiOBr | [IBU] = 50 mg·L^-1^ in UW,  catalyst dose = 0.5 g·L^-1^ | - | 5.5·10^-3^ | 1.49·10^-2^ | 4.55·10^-2^ | 2.04·10^-2^ | 2.23 | 55 |
|  | [IBU] = 50 mg·L^-1^ in DW,  catalyst dose = 0.5 g·L^-1^ | water matrix | 5.06·10^-3^ | 4.55·10^-3^ | 1.72·10^-2^ | 9.61·10^-3^ | 1.79 | 44 |
|  | [IBU] = 50 mg·L^-1^ in UW,  catalyst dose = 0.25 g·L^-1^ | catalyst dose | 5.36·10^-3^ | 5.60·10^-3^ | 2.16·10^-2^ | 1.09·10^-2^ | 1.97 | 49 |

^a^ in all tests: irradiance (when used) = 35 W·m^-2^, US continuous mode at 584 kHz, amplitude = 50%, when employed, whereas adsorption was carried out in the dark; ^b^ according to pseudo-first-order kinetics; ^c^ computed by eq. S.6; ^d^ computed by eq. S.7.

Figure S.10. Reactive species trapping results (scavenger-to-IBU molar ratio of 450:1).

Experimental conditions: IBU concentration = 50 mg·L^−1^; catalyst dosage = 0.5 g·L^-1^, US continuous mode at 584 kHz, amplitude = 50%, when employed.

Figure S.11. MS/MS spectra of the TPs and elucidation of chemical structures.

|  |  |
| --- | --- |
|  |  |
|  |  |
|  |  |
|  |  |
|  |  |
|  |  |
|  |  |
|  |  |

Figure S.12. Evolution profile of TPs in different degradation experiments.

Table S.6. *In silico* prediction of ecotoxicity, bioaccumulation and persistence of IBU and its TPs by ECOSAR software.

|  | **TAF^1^** | **TAD^2^** | **TAA^3^** | **TASW^4^** | **TAM^5^** | **TCF^6^** | **TCD^7^** | **TCA^8^** | **TCSW^9^** | **TCM^10^** | **BCF^11^** | **BAF^12^** | **LGP^13^** |
| --- | --- | --- | --- | --- | --- | --- | --- | --- | --- | --- | --- | --- | --- |
| **IBU** | 28.83 | 19.63 | 31.03 | 36.72 | 7.19 | 3.49 | 3.18 | 12.18 | 13.27 | 0.35 | 3.16 | 437 | 3.97 |
| **TP_121A** | 7.91 | 4.24 | 10.18 | 6.93 | 25.91 | 0.82 | 0.5 | 1.68 | 6.93 | 1.95 | 19.8 | 19.64 | 2.47 |
| **TP_121B** | 8.84 | 4.59 | 11.34 | 7.91 | 31.39 | 0.91 | 0.54 | 1.84 | 7.67 | 2.41 | 17.8 | 17.29 | 2.4 |
| **TP_159** | 1.85 | 4.44 | 0.11 | 35.68 | 16.01 | 0.28 | 0.17 | 0.7 | 6.1 | 1.11 | 3.16 | 8.59 | 2.74 |
| **TP_163** | 34.33 | 91.4 | 2.58 | 2280 | 1860 | 10.93 | 5.12 | 17.91 | 227 | 168 | 3.16 | 7.72 | 1.86 |
| **TP_165A** | 618 | 201 | 763 | 746 | 726 | 57.4 | 19.7 | 96.98 | 468 | 757 | 3.16 | 2.75 | 1.37 |
| **TP_165B** | 618 | 201 | 763 | 746 | 726 | 57.4 | 19.7 | 96.98 | 468 | 757 | 3.16 | 2.88 | 1.37 |
| **TP_171** | 164 | 568 | 23.41 | 256000 | 972000 | 350 | 89.65 | 195 | 6370 | 172000 | 3.16 | 0.92 | -0.41 |
| **TP_191** | 4200 | 2280 | 1420 | 5280 | 5400 | 390 | 197 | 337 | 429 | 537 | 3.16 | 3.8 | 1.53 |
| **TP_221** | 402 | 328 | 157 | 1360 | 850 | 37.6 | 57.78 | 84.81 | 172 | 68.14 | 101 | 236 | 2.25 |
| **TP_237A** | 40.72 | 229 | 26.11 | 443 | 187 | 19.75 | 81.51 | 3.95 | 79.88 | 12.64 | 3.16 | 8.59 | 2.83 |
| **TP_237B** | 1.5 | 1.21 | 2.33 | 443 | 187 | 0.07 | 0.72 | 3.06 | 79.88 | 12.64 | 3.16 | 8.59 | 2.83 |
| **TP_237C** | 40.72 | 229 | 26.11 | 443 | 187 | 19.75 | 81.51 | 3.95 | 79.88 | 12.64 | 3.16 | 8.59 | 2.83 |
| **TP_237D** | 1.5 | 1.21 | 2.33 | 443 | 187 | 0.07 | 0.72 | 3.06 | 79.88 | 12.64 | 3.16 | 8.59 | 2.83 |
| **TP_237E** | 40.72 | 229 | 26.11 | 443 | 187 | 19.75 | 81.51 | 3.95 | 79.88 | 12.64 | 3.16 | 8.59 | 2.83 |
| **TP_237F** | 40.72 | 229 | 26.11 | 443 | 187 | 19.75 | 81.51 | 3.95 | 79.88 | 12.64 | 3.16 | 8.59 | 2.83 |
| **TP_280** | 6510 | 3530 | 2170 | 8170 | 8530 | 602 | 302 | 512 | 652 | 856 | 3.16 | 2.75 | 1.5 |
| **TP_295** | 178 | 1440 | 66.17 | 3630 | 2850 | 98.96 | 540 | 8.75 | 375 | 252 | 3.16 | 2.75 | 1.92 |

^1^ Acute Toxicity Fish (LC_50_, 96 h) in freshwater; ^2^ Acute Toxicity Daphnid (LC_50_, 48 h); ^3^ Acute Toxicity Green Alga (EC_50_, 96 h); ^4^ Acute Toxicity Fish (LC_50_, 96 h) in saltwater; ^5^ Acute Toxicity Mysisd (LC_50_, 96 h); ^6^ Chronic Toxicity Fish (ChV) in freshwater; ^7^ Chronic Toxicity Daphnid (ChV); ^8^ Chronic Toxicity Green Alga (ChV); ^9^ Chronic Toxicity Fish (ChV) in saltwater; ^10^ Chronic Toxicity Mysisd (ChV); ^11^ Bioconcentration Factor;

^12^ Bioaccumulation factor; ^13^ Octanol/water partition coefficient.

Figure S.13. Principal component analysis (PCA) results of the ecotoxicological endpoints for IBU and its TPs: a) score plot and b) loading plot of the first two PCs. The variable labels in the loading plot are given in Table S.6.

Table S.7. Assumptions and computed parameters for preliminary economic evaluation on high-frequency US reactor (584 kHz, continuous mode, 50% amplitude).

| **General assumptions** | |
| --- | --- |
| Production unit = 1 m^3^·h^-1^  Work time = 6000 h·year^-1^  Equipment lifetime = 5 years  Discount rate: 8% → CRF = 0.251  Industrial electricity cost: 0.15 €·kWh^-1^  Lamp: 300 W  Chiller: 200 W  Catalyst dosage: 0.5 kg·m^-3^, 95% recovery (5% loss)  BiOBr cost: 32 €·kg^-1^  Pollutant: IBU, used only for testing, not included in industrial OPEX | |
|  | |
| **Initial investment (CAPEX, €)** | |
| High-frequency reactor (plate) | 18,000 |
| Lamp + power supply | 350 |
| Chiller | 2,000 |
| Dark box, accessories | 200 |
| **Total CAPEX *per* line** | **20,550 €** |
|  |  |
| **Annual CAPEX (5 years, CRF = 0.251) = 20,550 · 0.251 = 5,150 €·year^-1^** | |

| **Annual energy consumption (Hourly power demand (from lab data, scaled to 1 m^3^·h^-1^)** | |
| --- | --- |
| Ultrasound | 80 W (= 0.08 kW) |
| Lamp | 0.30 kW |
| Chiller | 0.20 kW |
| (Total) | 0.58 kW |
| **Total (0.58· 6000 h)** | **3480 kWh ≈ 520 €·year^-1^** |

| **Maintenance and spare parts** | |
| --- | --- |
| Plate (1000 € / 5000 h) → 0.20 €·h^-1^ · 6000 h | 1,200 €·year^-1^ |
| Generator (3% CAPEX) | 540 €·year^-1^ |
| Lamp | 900 €·year^-1^ |
| Chiller | 100 €·year^-1^ |
| **Total** | **≈ 2,740 €·year^-1^** |

| **BiOBr catalyst make-up** | |
| --- | --- |
| Dosage | 0.5 kg·m^-3^ |
| Loss (5%) | 0.025 kg·m^-3^ |
| Annual treated volume | 6000 m^3^ |
| BiOBr consumption | 150 kg·year^-1^ |
| **Total cost (150 · 32 €·kg^-1^)** | **≈ 4,800 €·year^-1^** |

| **Total annual costs** | |
| --- | --- |
| CAPEX amortization | 5,150 € |
| Energy (OPEX) | 520 € |
| Maintenance/spares (OPEX) | 2,740 € |
| BiOBr make-up (OPEX) | 4,800 € |
| **Total annual cost**  **with specific treatment cost (13,210 / 6000)** | **13,210 €**  **2,20 €·m^-3^ (COO)** |
|  |  |

Figure S.14. Comparative view of innovation potential in terms of COO of different US-based technologies. Data for horn, flow-through US and hydrodynamic cavitations comes from Ref. [3].

**References**

[1] E. Falletta, R. Bernasconi, V. Fabbrizio, E. Marcolini, A. Giordana, I.B. Iliev, L. Magagnin, C.L. Bianchi, Electrochemical treatment coupled with solar light-driven photocatalytic approach: A challenging process in cascade for hydrogen production and wastewater remediation, Journal of Environmental Chemical Engineering 12(4) (2024) 113139. https://doi.org/10.1016/j.jece.2024.113139.

[2] M. Sansotera, F. Persico, C. Pirola, W. Navarrini, A. Di Michele, C. L. Bianchi, Decomposition of perfluorooctanoic acid photocatalyzed by titanium dioxide: Chemical modification of the catalyst surface induced by fluoride ions, Applied Catalysis B: Environmental 148-149 (2014) 29-35. https://doi.org/10.1016/j.apcatb.2013.10.038.

[3] M. G. Galloni, V. Fabbrizio, R. Giannantonio, E. Falletta, C.L. Bianchi, Applications and applicability of the cavitation technology, Current Opinion in Chemical Engineering 48 (2025) 101129. https://doi.org/10.1016/j.coche.2025.101129.
